# Promising New Horizons in Medicine: Medical Advancements with Nanocomposite Manufacturing via 3D Printing

**DOI:** 10.3390/polym15204122

**Published:** 2023-10-17

**Authors:** Nan Li, Sadaf Bashir Khan, Shenggui Chen, Wurikaixi Aiyiti, Jianping Zhou, Bingheng Lu

**Affiliations:** 1 School of Mechanical Engineering, Xinjiang University, Urumqi 830017, China; dglgln@163.com (N.L.); bhlu@mail.xjtu.edu.cn (B.L.); 2 School of Manufacturing Science and Engineering, Key Laboratory of Testing Technology for Manufacturing Process, Ministry of Education, Southwest University of Science and Technology, Mianyang 621010, China; 3 School of Education (Normal School), Dongguan University of Technology, Dongguan 523808, China; 4 School of Art and Design, Guangzhou Panyu Polytechnic, Guangzhou 511483, China; dgutchensg@163.com

**Keywords:** tissue engineering, nanocomposite, drug delivery systems (DDS) medicine, 3D printing, stereolithography, fused deposition modeling (FDM)

## Abstract

Three-dimensional printing technology has fundamentally revolutionized the product development processes in several industries. Three-dimensional printing enables the creation of tailored prostheses and other medical equipment, anatomical models for surgical planning and training, and even innovative means of directly giving drugs to patients. Polymers and their composites have found broad usage in the healthcare business due to their many beneficial properties. As a result, the application of 3D printing technology in the medical area has transformed the design and manufacturing of medical devices and prosthetics. Polymers and their composites have become attractive materials in this industry because of their unique mechanical, thermal, electrical, and optical qualities. This review article presents a comprehensive analysis of the current state-of-the-art applications of polymer and its composites in the medical field using 3D printing technology. It covers the latest research developments in the design and manufacturing of patient-specific medical devices, prostheses, and anatomical models for surgical planning and training. The article also discusses the use of 3D printing technology for drug delivery systems (DDS) and tissue engineering. Various 3D printing techniques, such as stereolithography, fused deposition modeling (FDM), and selective laser sintering (SLS), are reviewed, along with their benefits and drawbacks. Legal and regulatory issues related to the use of 3D printing technology in the medical field are also addressed. The article concludes with an outlook on the future potential of polymer and its composites in 3D printing technology for the medical field. The research findings indicate that 3D printing technology has enormous potential to revolutionize the development and manufacture of medical devices, leading to improved patient outcomes and better healthcare services.

## 1. Introduction

Recently, 3D printing has become widely recognized as a potentially revolutionary innovation in medical fields. This technological advancement possesses the capacity to fundamentally reshape the manufacturing procedures associated with prosthetics and prostheses, thereby holding immense promise for the future of this field [1]. Additive manufacturing (AM), commonly known as 3D printing, is a process that allows for the rapid prototyping of nearly any structure, no matter how complicated, in a wide variety of materials [2]. Personalized medication distribution systems, anatomy models for surgery planning and training, and patient-specific medical prostheses are just a few medical uses that could benefit from this technology. The field of tissue engineering and regenerative medicine has benefited greatly from creating safe and bioresorbable materials for 3D printing [3]. Furthermore, the advent of 3D printing holds the potential to revolutionize the realm of personalized medicine, wherein individuals are bestowed with tailored healthcare interventions meticulously crafted to align with their distinct anatomical and physiological attributes [4]. This has the potential to improve patient results while also lowering healthcare expenses. The medical field presents its unique obstacles to 3D printing’s widespread adoption. Some problems that need fixing include creating better materials and methods, fine-tuning the printer settings, and standardizing the testing and evaluation procedures. As an added complication, 3D-printed medical gadgets and prostheses still need thorough testing and confirmation before the relevant governing bodies can approve them [5], i.e., 3D-printed renal models help in understanding patients’ renal anatomy [6], orthopedics and maxillofacial reconstruction in cardiovascular and liver diseases [7,8], or pre- and intra-surgical planning in kidney tumor treatment [9].

Despite the myriad of challenges that have impeded the realization of 3D printing’s utmost capabilities within the realm of medicine, diligent endeavors in research and development are currently being undertaken to effectively tackle these obstacles. Patient results, healthcare expenses, and the speed with which medical gadgets and prostheses can be designed and manufactured are just some of the many areas where this technology could have a positive impact [10]. Three-dimensional printing can also alter the structure and composition of materials throughout the life of a produced object [11]. It is possible to design thin-diameter lines and lattices, patterned dots, layers and porous membranes, and depth-changing motifs in functionally graded systems and intricate designs, such as in bone and nacre [12,13]. Using customizable materials allows for the control of new topology, form optimizations, foam density, and surface roughness [14]. Material modifications can be effectively accomplished using 3D printing (3DP) technologies, specifically material and binder jetting. In contrast, others, such as material extrusion and ink writing, allow for discrete control inside or between layers. Three-dimensional printing has several benefits over conventional manufacturing techniques, especially in medicine [15].

Recently, melt mixing has been employed to incorporate biocompatible polycaprolactone (PCL) and polyvinyl chloride (PVC) via 3DP using fused filament fabrication (FFF) [16]. Experimental tests reveal that PVC–PCL compounds are miscible due to high molecular compatibility and strong interaction. This causes extraordinary mechanical properties, especially for PVC–10 wt% PCL. In addition to the desired tensile strength (45 MPa), this material has an entirely rubbery behavior at an ambient temperature, and its total elongation is more than 81%. Programming tests show that PVC–PCL blends have an excellent shape memory effect, resulting in 100% shape recovery [17]. FFF 3D printers are expected to revolutionize the applications of PVC compounds in biomedical 3D and 4D (four-dimensional) printing due to their appropriate thermomechanical properties, supreme printability, and excellent biocompatibility [18]. Besides 3DP, 4D is also progressing, a novel approach to and design for fabricating two-layer composite structures, having a shape memory effect using a fused deposition modeling (FDM) technique with TPU elastomer filaments and the well-known thermoplastics ABS and PCL. The elastomeric layer is constrained by the thermoplastic layer. It is possible to stabilize the temporary shape and store the deformation stress for later recovery of the permanent shape by phase-changing the thermoplastic layer in the opposite direction during a rubber-to-glass transition of the thermoplastic layer. Findings demonstrate that the fixity and recovery rates of ABS–TPU were over 90%. The persistence of the PCL–TPU composite structure was 77.42%, but it also showed complete recovery [19]. These 3D/4D-printed shape memory devices are useful in the medical domain, i.e., in personalized endoluminal devices [20] or smart implants pre-designed to respond to (endogenous or exogenous) stimuli and perform seamless integration with regular/irregular tissue defects [21] or defect-luminal organs [22,23].

Three-dimensional printing allows the creation of implants and other medical tools uniquely suited to each patient based on their morphology and physiology [24]. By reducing the number of necessary surgical procedures and increasing patient satisfaction, personalization has the potential to boost clinical results while decreasing healthcare costs [25]. It helps in creating intricate shapes that would be challenging or impossible to start with more conventional manufacturing techniques [26]. In 2023, a patient’s mandibular morphology was recorded using a cone-beam computed tomography (CBCT) scan, and a virtual model was built using CAD software (AutoCAD 2022 [Version 24.1]) [27]. An implant-supported fixed prosthesis was subsequently designed using this concept. The SLS method is used to 3D-print biocompatible polyamide prosthesis and tissue regeneration [28]. Besides this, 3D printing generates reduced waste. Subtractive production processes such as cutting and milling are notorious for producing large amounts of unwanted scrap [29]. Three-dimensional printing minimizes waste by creating structures layer by layer. Prosthetics production is one practical application of 3D printing for waste reduction [30]. Traditional methods of producing prosthetics require subtractive processes such as cutting and milling, which generate a substantial amount of waste. In addition, 3D printing makes it feasible to design implants that have been tailored to an individual’s anatomy, enhancing a device’s fit and comfort. This customization can reduce the need for additional fittings and adjustments, reducing waste and related costs even further [31]. Furthermore, 3D printing allows for the creation of prosthetics on demand, reducing the need for pre-made device stockpiling and storage. This can result in expense savings and less waste in the supply chain. Three-dimensionally printed medical implants speed up therapy and improve outcomes [32], comprising metallic implants [33] or bone replacement implants [34].

Designing to imitate requires a hierarchical organization that demands a printing process and material selection that works well with the application. Structural integrity is necessitated using polymers based on methacrylic acid to maintain biocompatibility and allow for tissue development, i.e., heart valve prosthesis via TE [35]. Stereolithography printing has been employed to produce the intricate hierarchical truss structure required for the printing process. As a result of ambiguity and component variation in the 3DP process, it is critical to replicate and fine-tune the structure’s configuration. There are many different ways to create tissue scaffolds, depending on the material and printing process that is being employed. It is possible to generate PCL-based tissue scaffolds with a more malleable, biodegradable structure using fused deposition modeling [36]. The use of 3D printing technology to generate patient-specific medical equipment and prosthetic devices, biological models for operative preparation and training, and medication catheters is not an exception to this transformation in the medical industry. Polymers and the corresponding composites are growing increasingly popular in the healthcare sector as an outcome of their distinguishing thermal, mechanical, electrical, and spectral characteristics. This is especially helpful when it is impossible to precisely match the unique anatomical structure of each patient using traditional manufacturing techniques [37,38].

Complicated configurations that are typically challenging to generate using the traditional techniques of manufacturing can now be manufactured via the use of 3D printers. The ultimate result is a medical device that exhibits enhanced efficacy and user satisfaction. Prosthetic devices are manufactured using polymer materials and composites as well [39]. Prostheses have two fundamental purposes: they may either serve as substitutes for missing body parts or enhance the functioning of existing ones. Both functional and aesthetically pleasing prostheses may now be manufactured thanks to the use of 3D printing technology. The customization of the mechanical characteristics of polymers and their composites enables the fulfillment of specific patient needs. Producing anatomical models, medical devices, and prosthetics using 3D printing techniques is performed mostly for medical intervention planning and training. These models let doctors view a patient’s anatomy before treatment. This helps surgeons create a better surgical strategy and reduces risks [40]. Besides this, medical students have hands-on practice using anatomical models.

**Figure 1:**
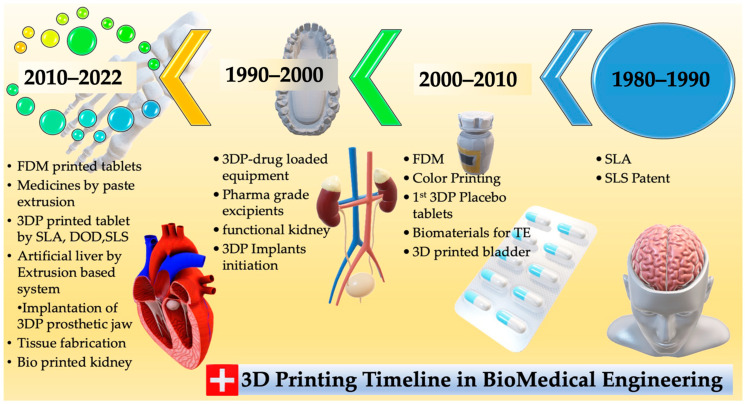
From the 1980s to the present, this timeline shows the history of 3D printing and its most important advances.

**Figure 2:**
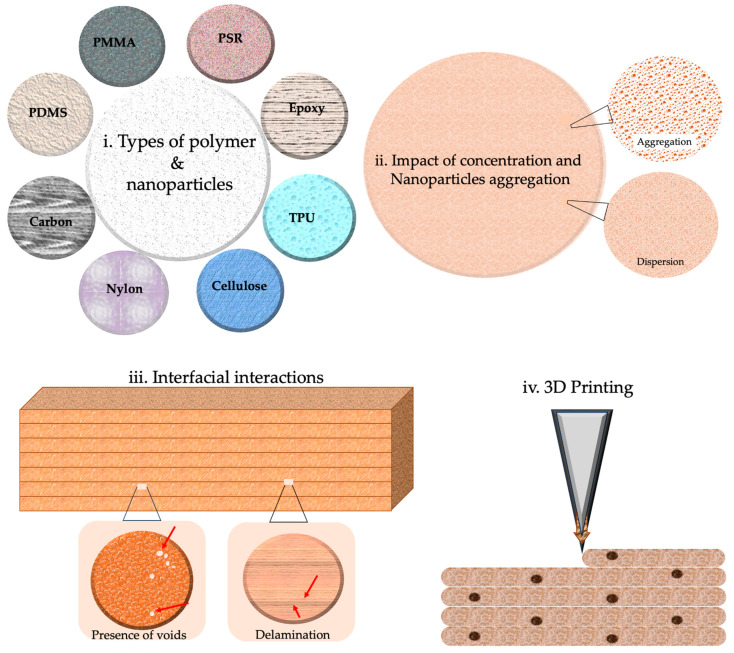
Critical parameters influencing 3D printing.

Figure 1 shows 3D printing’s early developmental stage in the 1980s, primarily for small product manufacturing or prototypes. New technical applications have been created since 2009, thanks to rapid development. Figure 2 shows AM discoveries and accomplishments. However, complex, high-precision, multi-material components are still in their infancy. The applications of 3D printing technology using polymers and their composites in the medical area are expanding rapidly, thanks to continued research and development. This is especially helpful for emergencies, in which delays in treatment can have severe consequences. Three-dimensional printing allows for rapid prototyping and optimization by producing multiple design iterations rapidly and cheaply. This can accelerate the creation of better medical implants and devices. This article presents a comprehensive exposition of the utilization of 3DP technology within the realm of medicine. An in-depth exploration is undertaken to analyze the prospective trajectory of 3DP within the realm of medicine alongside a comprehensive evaluation of the merits and demerits associated with employing diverse 3DP techniques for medical applications. This piece aims to aid the ongoing efforts to transform healthcare through this cutting-edge technology by summarizing the current state of the art in 3D printing for medical uses.

## 2. Polymer Particle Polymerization

A type of 3D printing known as polymer particle polymerization (PPP) uses polymer particles as building elements to construct intricate three-dimensional structures [41]. In PPP, a new structure layer is created by polymerizing a monomer around an already existing polymer molecule. Due to its capacity to produce intricate structures with a high resolution and a degree of control over the end product, this technique has grown in popularity recently, i.e., in the 3DP of carbon fiber [42]. Using tiny polymer particles as building elements in PPP enables the construction of structures with a high resolution. The method enables fine control over the finished product, including the mechanical properties, shape, and size. PPP has the capability to fabricate a diverse range of intricate structures, involving hollowed frameworks, permeable structures, and structures characterized by complex geometries [43,44,45].

Epoxy resins, nanoparticles, and polymeric or mixed materials may be manufactured using 3DP. Several factors, including heat, stress, moisture, the photopolymerization rate, the crosslinking phenomenon, corrosive chemicals, and UV light, can influence the properties of polymer nanocomposites. Intricate physical and chemical processes solidify a liquid monomer solution. When exposed to light, a photoinitiator in a solution breaks down into active radicals, which causes polymerization. In order to create polymer nanocomposites, monomers attack unsaturated double bonds. As a result, polymer chains and molecular weight rise, thickening liquid polymer solutions and changing the properties of the resulting polymers. Equations for the first-order rate of chemical reactions compute species concentrations. The monomer conversion level depends on the volume shrinkage, glass transition temperature, and nonlinear viscoelasticity of cured polymers. Mechanical properties should preferably be adjusted during photopolymerization to produce optimal strength and stability in the 3DP structure [46].

Frameworks, thermal transfer, electromechanical micro-machines, optoelectronics, surface changes, and biomedical applications can be enhanced by incorporating natural polymers with rigid body systems [46]. Polymeric materials are delicate, and most nanoparticles display inconsistency, a lack of adequate linkages with polymeric materials, or stiffness at large concentrations [47]. Increased filler aspect ratios in polymer matrices, for example, according to the shear lag hypothesis in composite dynamics, would boost the efficiency of stress propagation via fiber reinforcement, i.e., in carbon nanotubes [48]. The mechanical load cannot be transferred satisfactorily via discontinuous particles (nanowires or nanorods, i.e., ABS–ZnO nanocomposites) below a certain length, resulting in early fracture development before the composite collapses [49]. Composites’ performance may be improved via nano, micro, and macroscale particles [50]. However, a few key elements will decide the scope of remodeling, i.e., the kinds of polymer matrix and nanofibers, the dispersal or densities of nanomaterial, interfacial interactions, nanomaterial ordering, or the functional monomer [51,52]. Concerning high-performance nanocomposite polymer composites from 3DP technologies, herein, we will focus on three aspects, nanocomposite kinds, colloidal and initial concentration, and their interactions, as shown in Figure 2. The subsequent sections will provide a more comprehensive analysis of each of these aspects.

### 2.1. Polymer Nanocomposites: The Synergistic Combination of Polymers and Nanoparticles

Polymers and nanoparticles in 3DP have generated considerable attention due to their capacity to augment mechanical, thermal, and electrical properties [53]. Due to their intrinsic biocompatibility and capacity to undergo dissolution inside the human body, biodegradable polymers are often employed in 3DP, i.e., in polyglycolic acid (PGA), polycaprolactone (PCL), and polylactic acid (PLA) [54]. Scaffolding, implants, and systems for the administration of medicine are all examples of common applications for their use in the medical industry. Highly resilient polymers with exceptional thermal stability, specifically designed for employment in demanding sectors such as the aerospace and automotive industries, find their utility in the realm of 3D printing, where the need for materials capable of withstanding elevated temperatures is paramount. They comprise polyether ether ketone (PEEK) and polyphenylene sulfide (PPS) [52], while toughened polymers include acrylonitrile butadiene styrene (ABS) and polypropylene (PP) [53].

Similarly, metal nanoparticles such as Cu, Au, and Ag can be used in 3DP to enhance components’ thermal and electrical conductivity [54]. They are frequently employed in the creation of sensors and electronic gadgets [55]. Adding polymeric nanoparticles to 3D printing may boost the thermal, mechanical, and electric attributes of the generated components, i.e., with polyhedral oligomeric silsesquioxane or graphene oxide (GO) [56]. Ozbolat and Hospodiuk (2016) examined the applications of 3D printing in tissue engineering using biodegradable polymers and composite materials [57]. Three-dimensional printing was used by Shi et al. (2017) to create thermoplastic composites with exceptional performance characteristics through the inclusion of carbon nanotubes [58]. Shukla and his colleagues (2019) developed polymers reinforced with graphene via 3DP, enhancing their mechanical and thermal properties [59]. In addition to liquid polymerization, nanoparticle-suspended particles, and epoxy formulations, 3D printing (3DP) has the potential to operate using various other materials such as copolymers, suspensions, polymeric slurries, and continuous thin sheets [60].

The enhancement of the mechanical qualities of composite materials may be achieved by combining the inherent features of these materials with those of their component phases. The kind of polymer and nanoparticle utilized has an impact on processing parameters, material structures, and system properties [61]. This implies that conditions for 3D printing will be defined by diverse epoxy resins, as well as ultra-high-performance polymers. For example, vat polymerization is ideal for employing light-curable polymers [62]. Material jetting may use low-viscosity inks to obtain fine details. On the other hand, printed green components have poor mechanics and have been best utilized in functional materials, rather than structural materials, since they have weak mechanics [63]. Extruded filaments may be made from various materials for wound membrane use, but their use in fast prototyping and tissue scaffolds is restricted [64].

### 2.2. Nanoparticle Dispersions and Concentrations

Nanoparticle dispersions and concentrations can greatly impact the properties of 3DP parts [65]. The mechanical characteristics of printed parts are influenced by the distribution of nanoparticles within a polymer matrix, whereas the concentration of nanoparticles influences the thermal and electrical properties, i.e., in Ti_3_C_2_/epoxy nanocomposites [66]. A homogeneous dispersion of nanoparticles within the polymer matrix may enhance the mechanical properties of printed components. This can be accomplished using a variety of methods, such as sonication, ultrasonication, and surfactant-assisted blending [67]. Aggregating nanoparticles can cause the weakening of the mechanical properties of printed components within a polymer matrix. This can be prevented by evenly dispersing the nanoparticles and avoiding agglomeration during printing. The properties of printed components can only be slightly altered by low concentrations of nanoparticles in a polymer matrix [68]. However, nanoparticles can enhance the mechanical qualities of printed parts even at modest concentrations. High nanoparticle concentrations within a polymer matrix can enhance printed components’ thermal and electrical properties. However, a concentration that is too great might aggregate and have weaker mechanical properties [69]. Nanoparticle dispersions and concentrations are essential factors in 3DP because of their impact on the mechanical, thermal, and electrical properties of printed objects [70]. By creating a homogeneous dispersion of nanoparticles inside a polymer matrix and selecting an appropriate concentration, the qualities of printed components may be enhanced, i.e., in natural fiber-reinforced PLA or propylene composites [70,71]. Another technique for preventing particle aggregation is to regulate the external fields around the particles [72]. Electrophoresis is a method used for the efficient alignment and dispersion of particles inside a solution via an electrical field to scatter and arrange particles [73]. This high electrical voltage may result in the formation of electrical percolation spots, electricity generation intensities, and electrostatic interactions, all of which may lead to the formation of nanoparticle agglomerates [74]. The intensity of magnetic fields affects both particle density and dispersion in a similar manner to the way electric fields’ strength does. Thus, the presence and concentration of nanoparticles in the polymer matrix significantly impact 3DP resolution [75].

### 2.3. Interfacial Interactions at the Polymer–Particulate Interface

Understanding and optimizing the interfacial interactions at the polymeric–particulate interface is critical in creating high-performance and functional composite materials for tissue engineering, drug delivery, and surgical planning [76]. When nanoparticles or microspheres are incorporated into a polymeric matrix to form a composite material, interfacial interactions occur at the polymeric–particulate interface [77]. These particles can improve printed components’ mechanical strength, biocompatibility, and drug delivery capacity. Interfacial adhesion and printed component performance might suffer if the particles and polymer matrix fail to interact effectively. Multiple variables may influence these interactions, including surface chemistry, particle dimensions, morphology, concentration, dispersion, and the circumstances of 3D printing processes [78]. Regarding 3DP nanohybrids, interfacial interactions occur at the polymeric–particulate interface, within printing inter-lines, or between inter-layers [79] because an interaction involving polymeric materials and nanoparticles transmits stresses from tougher nanoparticles to softened polymers [80].

**Figure 3:**
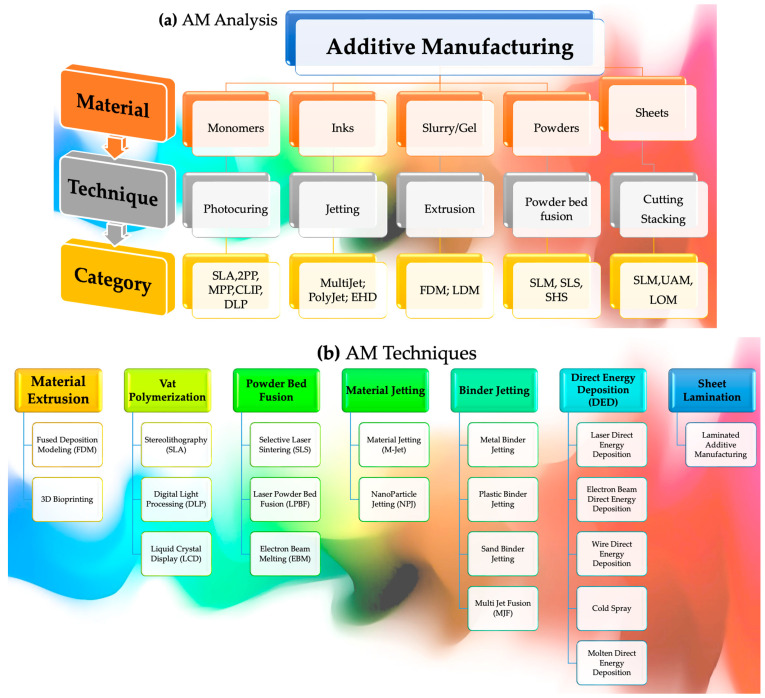
Overall progression displaying (**a**) AM analysis comprising numerous techniques and categories alongside (**b**) AM methods.

Morphologies disclose the kind of bonding and fracture that has occurred, i.e., pull-out, delamination, or fiber breakage. Thermomechanical investigations, residue stress mapping, and numerical micro/nanomechanical testing can be utilized to acquire a deeper understanding of the interactions among polymers and nanoparticles at the interface [81]. These approaches demonstrate how these interactions influence material characteristics. Interfacial interactions between polymers and particles, as opposed to interlayer bonding modification, are more likely to include the chemical alteration of the polymer [82]. Improving hydrophobic particles’ ability to adhere to hydrophilic polymers is important, such as in PVA and PEG [83]. The oxidation process can add functional entities (such as C–O, O–C–O, and –NHCO–), as well as bonds (for example, N–H, O–H), into these particles, influencing the surface tension and interactions [84]. Using enzyme treatment, natural fibers’ hydrophilic surfaces become compatible with hydrophobic polymers, allowing them to be used together. Polypropylene (PP) is an excellent material for the manufacturing of composite materials, owing to its notable characteristics, including its low surface tension and chemical inertness [85]. Using a maleic anhydride coupling agent in conjunction with PP grafting on maleic anhydride may help to improve the binding between polypropylene and glass fibers [86]. Inter-line bonding and inter-layer adhesion are less complicated to investigate than nanoscale polymer/particle interactions, which are more difficult. In the absence of voids, efficient adhesions may prevent fracture. Nozzle temperature and foundation impact bonding neck diameter and volume; for example, they affect interfacial bonding in a significant way [87]. Both the material used and the circumstances under which it is processed impact interlamellar fracture toughness and shear strength. The kinetics of polymer crystallization may be altered by printing layers that are heated and cooled at different periods, resulting in poorer bonding between neighboring lines between successive printing layers. It has been shown that objects manufactured utilizing FDM, SLS, or inkjet exhibit lower-yield ultimate tensile stress along the *z*-axis versus materials produced by employing traditional manufacturing procedures in several circumstances [88]. Improved interlayer bonding may be achieved in various situations using an increased temperature to cause polymer liquefying, lower printing rates to produce fewer flaws, and a lowered width thickness to avoid porous formation, among other things [89]. The adhesion and particle dispersion rates may be varied by altering the materials used in these products. Figure 3 represents numerous AM techniques, materials, and the processing involved.

## 3. Light-Induced Polymerization for Advanced Materials

During the 3DP process, photosensitive monomers are exposed to high-intensity light sources, which causes them to degrade. Compared to other printing techniques, resin curing procedures often have greater resolutions and produce higher-quality products than other printing techniques. This has resulted in various methods of fabricating parts from resin vats exposed to ultraviolet light, such as stereolithography with direct laser writing. A laser operated via a computer hardens the resin in the tank, resulting in a solid coating layer. Following the exposure of each layer in the printing process, the printing platform advances vertically. To enhance the structure and enable fine-tuning to match the requirements of particular applications, the material is then cleaned and UV-cured. After a 30-h curing period, UV radiation is more effective and boosts mechanical qualities, such as the elastic modulus, while promoting material consistency via enhanced crosslinking. In stereolithography, printing may not always allow the simultaneous printing of several materials despite its high print resolution and speed. Polyjet printing, also known as inkjet printing, is an alternate resin curing technique that does not need ultraviolet light. It is possible to print multi-material objects in a short time using multi-nozzle jetting and support materials. Recently, inkjet printing can be applied to prototyping, electronics, and even bioprinting. Beams with diameters of around 400 nm have been employed to print lattices that exhibited fabrication faults due to the structure’s topology and build orientation. Although polyjet printing has implications in tissue engineering, more research is necessary to decide whether this process is suitable for assembling configurations that can support cell seeding and proliferation.

### 3.1. Powder Bed Fusion

Powder bed fusion (PBF) uses numerous materials and is a frequently used technique. The direct metal laser sintering (DMLS), electron beam melting (EBM), selective heat sintering (SHS), selective laser melting (SLM), multi-jet fusion (MJF), and selective laser sintering (SLS) printing processes are all included in the PBF process. A few examples of materials that are in use in PBF and demonstrate semicrystalline characteristics are ceramics, polyamides, and polyimides, in addition to metallic materials and titanium alloys [90]. Only a few preparations can produce pure polymer powder in large quantities, i.e., via the mechanical approach, which involves milling and grinding to make microscopic pellets in a cryogenic environment [91]. The solution technique depends on the thermally induced phase separation of a solution. Because of a change in temperature during this procedure, micro-sized particles of a polymer solution precipitate from the solution. Co-extrusion is a commonly used technique in the process of polymer melting. This technique involves melting a polymer extrusion and cooling it in a solvent, which allows the polymer matrix to be dissolved. The spherical particles can be recovered via filtering or screening. It is difficult to achieve a homogeneous distribution of discontinuous reinforcing fillers in SLS-produced polymer composites, which is a key challenge in producing polymer composites [92]. Spray drying, in situ polymerization, solution mixing, mechanical blending, and wet mixing are some methods for dispersing particles, similar to polymer powder technologies [93].

As far as mixing is concerned, mechanical blending relates to the method of merging epoxy resin and particulate granules in a dry condition by integrating them together with the help of a mechanical homogenizer or cryogenic milling as the method of linking [94]. Nanofibers, hydroxyapatite, and black carbon granules were manufactured using cryogenic milling, e.g., in nylon or PEEK matrices [95]. PBT/PC/MgO composite powders can be processed to particle sizes ranging from 20 to 100 microns that are consistent and well-controlled through ball milling [96]. The interaction between polymers, without the use of transesterification, may produce particles that are uniform in shape and have adequate bulk density and rheological properties. This usually leads to the crystallization of the mixture because the two kinds of particles have partly separated and agglomerated. The flexible nature of epoxy resins may also inhibit pellet formation when heated above their glass transition point (i.e., 50 °C). It is important to note that wet mixing differs from drying because it is centered on suspensions or nanoparticle surface coating on polymer granules. Van der Waals interactions, electrostatic forces, and other secondary bonds may aid the production of polymers and particle powders [97]. The use of low-viscosity solvents and a somewhat lower process temperature than is generally utilized may result in powders with a spherical shape. Polymers including PEEK, POM, PBT, and PS may have previously benefited from silica’s effective integration [98]. Carbon black adsorbed on the surface of PA12 particles may be converted into powders via mechanical sonication and mixing that can then be filtered. The shape of these nanocomposites was spherical, and their size distribution was uniform throughout the specimen [99].

To disperse them, extruding particles in polymers during melt mixing is important. They are then reduced to fine particles via grinding or rotating shearing techniques. Ceramic particles such as Al_2_O_3_, Fe_2_O_3_, SiO_2_, or CoO are appropriate for inclusion in polymers such as PC, PVC, PE, and polyamidoethylene terephthalate [100]. Micro-sized particles may be more successful in dispersion than nano-sized fillers in polymer melts, partly due to the polymer melts’ high viscosity. Solution mixing dissolves polymer–particle combinations in a solvent before precipitation or solvent evaporation. Ding H et al. produced PEEK/PPS and ketone particles via thermally induced phase separation [101]. They noticed that the powders generated were virtually spherical and had consistent size distributions and smooth flowability, indicating high quality. In most circumstances, the solution mixing of CNT in TPU is more likely to result in better particle dispersion than melt mixing despite being less viscous [102]. In the physicochemical field, spray drying involves applying pressure to an atomizer needle to sprinkle a solution’s droplets over a surface [103]. The solvent evaporates instantly, resulting in the formation of polymer nanocomposite particles. Although the particle sizes are usually too tiny for SLS processing, this is often due to the particles’ small size. This has resulted in composite powders being sprayed in less often than they could have been in the past. The particle size when spraying was 26 microns, but the particle size after milling was 52 microns [104].

Monomers and initiators accelerate polymerization. Particle dispersions are improved, and the adsorption and coating of polymer chains on particle surfaces are enhanced. PVP on PA12 surfaces exhibits higher adsorption and coating [105]. Some polymer–particle hybrid powders have the ability to boost the interactions between their constituents drastically in nickel–PA12 composite [106]. When using a high concentration of reinforcing fillers, the viscosity may increase throughout the sintering process, but increasing laser intensity may enhance flow dynamics. A severe laser pulse must be prevented because of the disintegration of polymer particles that occurs due to high laser intensity. Thermal and residual stress will be generated throughout the printing process as a medium of interaction between the light source and the polymeric material at various stages throughout the process. By analyzing the particles’ optical properties, one can figure out the capacity for absorption and reflection of the energy source and the degree of heat propagation and dissipation. This is performed using laser energy, which melts and fuses particles while simultaneously maintaining the operating temperature within the steady sintered range provided by the light source. As a result of the complexity of management and the difficulty in reaching high temperatures, which results in delayed diffusions, residual tension in printed goods is formed in the printed product manufacturing process [107]. It is required to treat the material after it has been heated since stress is dependent on both the variation in temperature and properties, including its melting point, diffusion kinetics, and surface tension.

### 3.2. Vat Polymerization

Photopolymerization, a 3D printing technology, relies on the same basic strategy: a liquid photopolymer contained in a vat (or tank) is selectively cured. VAT photopolymerization 3D printing includes stereolithography (SLA), digital light processing (DLP), and continuous liquid interface production (CLIP).

#### 3.2.1. Stereolithography (SLA)

SLA may be utilized to generate innovative composites that include ternary phases or have sensitive features, such as the capacity to absorb energy. More cured resins that are stiff yet brittle may be employed. It is conceivable to enhance their durability through the incorporation of particles endowed with the capacity to assimilate and dissipate energy. Core-shell particles with epoxide functional groups were produced by Li et al. These particles were subsequently observed to effectively adhere to SLA-printed epoxy resins [108]. An emulsion polymerization process was employed to generate an outer shell on the particles composed of a polybutadiene core and PMMA on the outside. The flexibility, durability, and damage tolerance of solid and cellular materials increased. Incorporating self-healing characteristics is another technique for minimizing the risk of brittle failure in SLA-printed epoxy. Beckingham et al. made a discovery about the enhancement of durability and material sustainability in SLA 3D-printed specimens. They found that, by integrating a self-healing microcapsule catalyst system with a readily available photosensitive polymer matrix, significant improvements were achieved [109]. Before the SLA 3DP self-healing composite, polymer microcapsules holding healing fluids were dispersed in resin. The specialized biological and therapeutic functioning of SLA enabled hierarchically ordered biomaterials. When used in place of a filler, reinforcement fillers may increase viscosity. Due to greater viscosity, the processing will be slowed down significantly. As UV light dispersion decreases the UV penetration depth and lateral resolution, it is not practical to employ higher laser powers or UV-reinforcing fillers. Due to localized overheating, the premature curing of polymer breakdown may occur. Adding additives to the resin/particle system may help polymerization, adjust the viscosity, preserve particle stability, and increase interfacial bonding among the particles.

**Figure 4:**
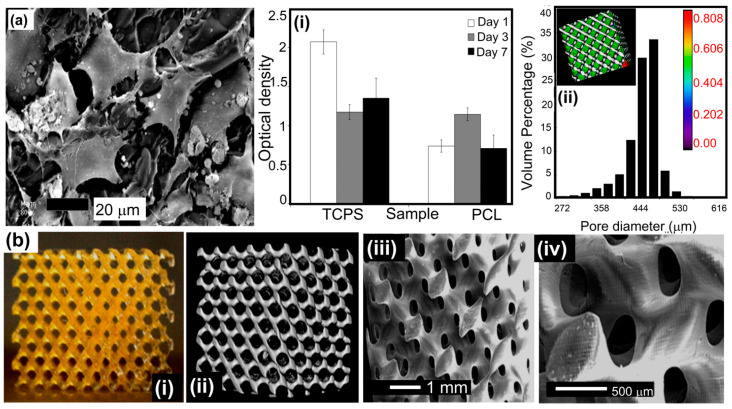
(**a**) SEM image: fibroblasts after 7 days in a cell culture showing crosslinked PCL, (**i**) the activity of cells’ metabolism while grown on TCPS (positive control) and photo-crosslinked PCL networks, and (**ii**) pore size distribution throughout the porous scaffold via μ-CT. (**b**) Photographs of (**i**) a micro-computed tomography visualization and (**ii**) SEM of a scaffold via SLS using a 1500-m macromer (**iii**,**iv**) An enlarged view of the open and linked nature of the porous structure indicates that the scaffold exhibited suitable external and internal surfaces. Reprinted with permission [111].

Several novel SLA-based methods, such as two-photon polymerization (2PP)/digital light processing (DLP) and multi-photon polymerization (MPP), have been developed [110]. The CLIP technique is an exception to this judgment since it is not extensively utilized in composites. SLA enables the dependable and repeatable production of a wide range of 3D structures since the final microstructure and form can be accurately controlled. Non-linear scaffold geometries may also be fabricated. Photo cross-linkable poly(e-caprolactone) (PCL)-based resin was produced and applied via stereolithography by Laura Elomaa and colleagues, as shown in Figure 4 [111]. The structure’s preparation did not involve the use of any additional solvents. Gel-rich networks were made by synthesizing PCL oligomers with three arms of varying molecular weights, functionalizing them via MAA anhydride, and then photo-crosslinking the resulting molecules. Stereolithography and a resin made of PCL macromer, the Irgacure 369 photoinitiator, an inhibitor, and a dye were used to make porous scaffolds. The resin was heated during curing until it achieved the proper viscosity. The scaffolds were a perfect replica of the CAD drawings with no evidence of material contraction. The sample porosity was determined to be 70.5 ± 0.8%, having an average pore size of 465 µm. There was a significant interaction between the pores. In tissue engineering, photo-crosslinkable and biodegradable PCL resin is ideal for creating scaffolds via solvent-free stereolithography. In a PLC sample, fibroblasts were seen after 7 days in culture., as shown in Figure 4a. Tissue culture polystyrene (TCPS) served as a standard in an MTS experiment measuring cellular metabolism at the film’s surface. The data are presented in Figure 4(ai,aii). Even after just one day, optical density, which correlates with the quantity of live cells, increased. By day 3, there was a striking resemblance between the optical density and the standard TCPS sample. The PCL networks’ surface area was completely populated by cells. The total number of viable cells dropped as the confluence reached 100%. In cytotoxicity assays, the photo-crosslinked networks made from a methacrylate PCL macromer and Irgacure 2959 photoinitiator were compatible with living tissue. Three-dimensional porous structures were fabricated using SLA at temperatures between 43 °C and 46 °C utilizing a resin made from the PCL 1500-m macromer. The resin had a photoinitiator, inhibitor, dye, and macromer. The scaffolds were very porous, with the pores fully interconnected across the three-dimensional structures. The samples’ exposed surfaces were smooth and homogeneous in quality. After curing, the scaffolds retained the same dimensions as before extraction and drying. That is why we did not see any evidence of material shrinking. The yellow color of the scaffolds, as described in Figure 4(bi), was due to triethylamine forming a colorful complex with MAA anhydride. In lCT reconstruction, the porous structure was found to be open and linked (Figure 4(bii)). The scaffold appeared to have appropriate external and internal surfaces in the SEM data (Figure 4(biii,biv)). The scaffolding was a precise representation of the CAD-drawn framework.

Organ printing uses tissue spheroids as key components to create living, functioning organs in a 3D configuration. Microtissues and tissue spheroids are living materials that can be studied and traced throughout time due to their unique composition, material, and biochemical characteristics. Tissue fusion is fundamental to the physical and chemical understanding of tissue self-assembly. Small, solid, and lumenized vascular tissue spheres can create tiny branches of an intraorgan vascular tree. This might open the path for the large-scale industrial robotic biofabrication of perfusable intraorgan branching vascular trunks in real human organ structures. Organ printing can potentially improve and alter the field of tissue engineering significantly. Consequently, organ manufacturing is a novel enabling technology that offers a developmental-biology-inspired replacement for the traditional biodegradable solid-scaffold-based techniques of tissue creation.

**Figure 5:**
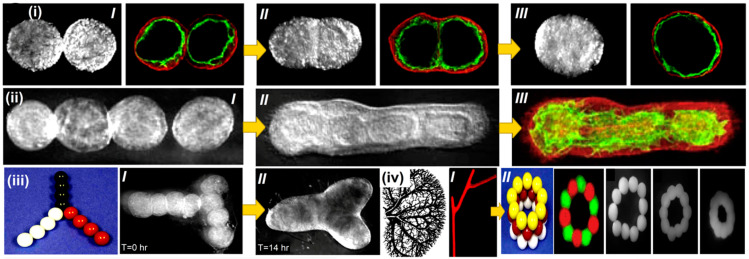
Bioprinting intraorgan branching vascular trees with uni-lumenal vascular tissue spheroids. Developmental stages of a ring-shaped vascular architecture during tissue fusion. (**i**) [*I–III*] Bioengineered vascular tissue spheroids in the shape of rings made from human smooth muscle cells. To show that no cellular mixing occurred during tissue fusion, tissue spheroids were fluorescently stained with green and red fluorescent stains, respectively. (**ii**) [*I*] Uni-lumenal vascular tissue spheroids fusing together in a hanging droplet. (**ii**) [*II**–**III*] Successive procedures for vascular tissue spheroids in collagen type 1 hydrogel fusing together. (**iii**) Physical representation of the development of branched vascular segments from spheroids of uni-lumenal vascular tissue in the production of type 1 collagen hydrogel (**iii**) [*I*] initially; and (**iii**) [*II*] after the integration of tissues). (**iv**) Kidney intraorgan vascular tree segment bioprinting employing solid vascular tissue spheroids. [*I*] A piece of a vascular tree that was bioprinted. [*II*] A bioassembly model of a tubular vascular tissue construct in 3D employing spheres of solid tissue. Reprinted with permission [113].

Vladimir Mironov and his colleagues aimed to describe and explore a fresh, rapidly emerging workflow in tissue engineering inspired by embryonic biology [112]. Anatomically precise tissue spheroids can be biofabricated in a lab. Some of the most crucial branches of a vascular tree have already been shown to be individually engineered. Additionally, in vitro and in vivo research shows that microvascular tree fragments as small as 100 nm can reassemble themselves, as shown in Figure 5(i–iii). As a result, it is feasible to build an intraorgan vasculature network having 10–12 orders of branches—specifically for organ printing technology, a bioengineered intraorgan branching vascular tree made up of three different types of self-assembled vascular tissue spheroids. The bioengineered vascular tree may be perfused and integrated into 3D tissue or organ structures after bioprinting, post-printed tissue fusing, and faster tissue maturation. Bioengineering a hierarchically branching intraorgan vascular tree for a bioprinted 3D tissue construct is challenging. It is hard to print functioning human organs without branching intraorgan vascular trees. The scholarly work conducted by C. M. Smith and colleagues delved into the realm of exploring the feasibility of employing three-dimensional direct-write cell deposition as a means of fabricating dynamic frameworks [113]. To facilitate the step-by-step assembly of cells and ECM on different substrates, a direct-write bioassembly device was developed and manufactured. In this experiment, human fibroblasts were coextruded onto a polystyrene slide through positive displacement delivery while suspended in a polyoxyethylene/polyoxypropylene mixture. Approximately 60% of the fibroblasts that were subjected to deposition exhibited viability even after the elapse of a 24-h period, as experimentally proved by Smith CM [113]. By employing a micro dispensing technique, the bovine aortic endothelial cells (BAECs) were coextruded onto the hydrophilic surface of polyethylene terephthalate sheets. Upon being introduced via a 25-gauge tip, a remarkable survival rate of over 86% was observed among the BAECs. The configurations survived in culture for up to 35 days while retaining their initial spatial arrangement. These findings show the possibility of a direct-write, 3D bioassembly strategy to create pattern-driven tissue-engineered structures. The model employed in these investigations was built from pig cardiac angiography. The construct had not yet undergone cell elongation and proliferation two hours after extrusion. The experimental results revealed proliferation, phenotypic differentiation, and pattern persistence when cultures were kept alive for up to 35 days [113].

#### 3.2.2. 2PP/MPP 3D Printing

It has been shown that 2PP or MPP, commonly known as direct laser writing (DLW), has a significant manufacturing capacity that may be used in biomedical engineering. When an initiator preferentially absorbs a single UV photon of a short wavelength containing monomers or oligomers, 1 PP takes place, as observed in traditional SLA, and polymer chain production begins to occur. The photosensitive resin absorbs ultraviolet rays within several micrometers of its layer width due to its low penetrating ability. The excitation laser wavelength for 2PP is much less than that of the excitation laser (when compared to one-photon polymerization (1 PP)). The multiphoton polymerization (MPP) process, which occurs during the photo-crosslinking of polymers, absorbs three or more photons simultaneously. CNT alignment in nanostructured resins was achieved via laser writing through 2PP. The changing power of the polymer/CNT curing laser (375–995 nm) affected the composite. This study hypothesized that reducing the nematic order parameter would benefit sensors, actuators, and metamaterials. The thiol-grafting strategy improved concentrations of thiol-acrylate composites by up to 0.2% of the CNT surface modification [114]. The CNT’s inclusion resulted in a significant improvement in mechanical and electrical properties. Achieving this CNT alignment on a large scale in scalable production is challenging. For example, no preferential orientation was observed because of the higher electrical conductivity of CNT cured in resins. Laser curing has also been shown to produce intriguing phenomena in ZnO/resin [115], Au nanorod actuation in liquid crystalline rubbers [116], and gold ion aggregates in SU8 resins [117].

**Figure 6:**
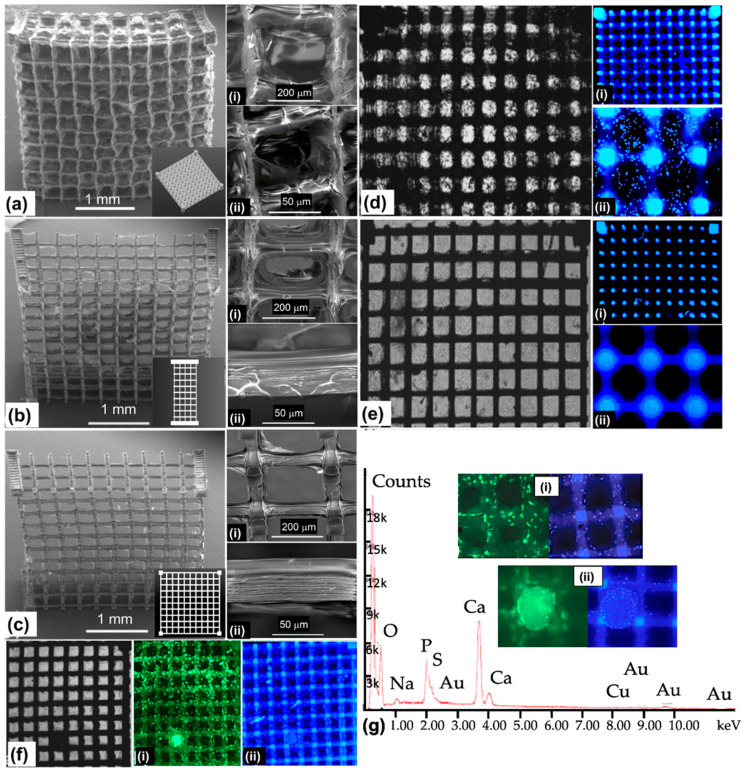
Gelatin scaffolds were created using the 2PP approach. Scaffold CAD model analysis and SEM images of (**ai**,**aii**) untreated scaffolds. Scaffolds using collagenase solution with 100 CDU/mL for (**bi**,**bii**) 1 h and (**ci**,**cii**) 2 h. Fluorescence images of MSC-seeded gelatin scaffolds (**di**,**dii**). High seeding efficiencies were achieved via cell entrapment on an untreated scaffold with mesh-filled pores, a (**ei**,**eii**) collagenase-treated scaffold, and (**f**) an 11-day-old porcine MSC-seeded gelatin scaffold. SEM image (**i**,**ii**). Blue and green fluorescence images show cell distributions. (**g**) EDX confirmed calcium and phosphate on a 2PP-produced scaffold. (**i**,**ii**) Calcium phosphate nodules, magnified view. Reprinted with permission [118].

Figure 6 illustrates the selected scaffold design’s top and side views. The scaffolds’ pore array was 10 pores deep and 10 pores wide (250 μm × 250 μm, 300 μm spacing) [118]. On each side of the scaffold, there were also four porous layers. The scaffold was constructed layer by layer in a CAD model with a slice distance of 15 m in the vertical axis. Parallel laser scans from a distance of 2 μm were used to build up each successive layer. To expedite the manufacturing of these massive scaffolds, the average laser power was increased to 3.5 mW, the highest permitted by the current setup. At this power, 2PP manufacturing at 10 mm/s scanning speed took 5 h per scaffold. Utilizing a more potent laser would aid in the reduction of production time. After three washes at 55 °C in distilled water to remove unpolymerized GelMOD, the final structure was uncovered. Prior to the SEM examination, the constructions were freeze-dried. The findings suggest that scaffold perforations have mesh-like patterns (Figure 6(ai,aii)). Researchers think diffusion-driven polymerization formed the mesh since the scaffold pores were not laser-irradiated. From the irradiated zone, laser-generated radicals disperse and induce polymerization. In accordance with the procedure used in material degradation investigation, incubating scaffolds in a collagenase solution (100 CDU/mL) resulted in partial (1 h incubation, Figure 6(bi,bii)) or total (2 h incubation, Figure 6(ci,cii)) meshes’ disintegration. Removing the excess mesh from the scaffold revealed its quality and dimensions. The analysis indicated that the polymer struts shrunk from 50 to 40 μm in width after the scaffolds were cured. When restored to an aqueous environment, the scaffolding expanded and regained its previous size. Figure 6(cii) shows that the polymer struts had linear micro patterns that are 1.5 μm apart. The patterns created via the scaffold manufacturing process resulted from a linear scanning technique. Considering shrinkage processes, the separation of this ridge-like microtopography correlates with the initial laser scanning settings utilized for 2PP manufacturing. Hence, 2PP allows for exact CAD model replication. Furthermore, 2PP affords control over a scaffold’s microtopography in addition to its porous structure and configuration. The engineered scaffolds’ biocompatibility was then assessed in vitro. The cells’ responses after mesenchymal stem cell (MSC) placement were examined to assess the scaffolds’ efficiency. The objective was to investigate the cell response and evaluate the potential usefulness of this technique in tissue engineering. The ability of collagenase-treated and untreated scaffolds with vacant pores and meshes to enable cell seeding was evaluated. To facilitate initial cell adhesion, the scaffolds were seeded with 4 × 10^4^ MSCs and then incubated at 37 °C. The additional medium was introduced to the scaffolds following a duration of 1 h, subsequent to which their progress in cultivation was observed and evaluated. A microscopic examination was conducted on scaffolds that had been seeded with cells one day following the seeding process.

The nuclei of the cells were stained with Hoechst 33342. The determination of cell density and scaffold localization may be inferred by analyzing the staining intensity and autofluorescence shown by the scaffold. Cell densities were found to be the maximum on polymer struts and within untreated scaffold pores when a mesh filled the pores (Figure 6(di,dii)). After 2 h in a collagenase solution, the scaffolds had opened pores. Thus, most cells were unable to adhere to the scaffolds after seeding (Figure 6(ei,eii)). Since the mesh within the pores maintains cells and promotes cell seeding efficiency, untreated scaffolds aid in seeding. Afterward, unprocessed scaffolds were cultivated in 1 mL of full DMEM at 37 °C in a humidified environment (95% air and 5% CO_2_). The addition of osteoinductive media followed three days of early cell growth. Calcein AM and Hoechst 33342 staining was performed on the cell-seeded scaffolds on day 11. The specimens were then examined by employing fluorescent microscopy. The use of Hoechst for cell localization is due to its ability to stain cell nuclei, while Calcein AM allows for the imaging of live cells and the examination of their adherence and shape. The scaffolds were found to be stable in culture media for 11 days.

There was also no significant swelling or distortion during culturing (Figure 6f). The fluorescence at several focal planes across the scaffold showed that cell migration was not inhibited by the mesh present in the pores after initial seeding. The cells were seeded all over the scaffold (Figure 6(fi,fii)). Calcium and phosphate were both detected through the EDX study (Figure 6g). The cells appeared to adhere and spread out uniformly across the polymer struts in the SEM micrograph (Figure 6(gi)). The presence of calcium phosphate nodules near osteoblast clusters indicated appropriately differentiated cells (Figure 6(gii)). The engineered scaffolds would be robust enough to resist an active cell culture, such as that carried out in a perfusion bioreactor. The mesh had a minimal influence on nutrition transport for the present scaffold size. Based on the degradation behavior, this mesh would break down and release the pores while leaving the rest of the scaffold intact. The results show that, with success, photopolymerizable GelMOD can be used with 2PP scaffolds for skeletal tissue engineering.

#### 3.2.3. 2PP: Conducting Polymers

**Figure 7:**
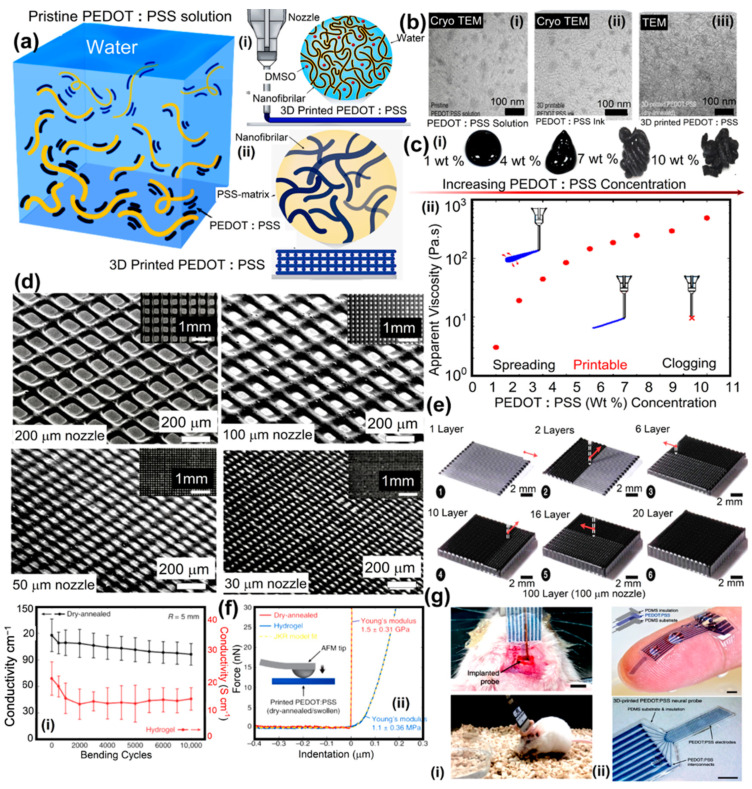
(**a**) Creation of a pristine PEDOT:PSS solution for (**i**) 3D-printed conducting polymer ink, (**ii**) using cryogenic lyophilization followed by re-dispersion in a solvent. (Dry annealing followed by swelling in a wet environment can transform the dry state of 3D-printed conducting polymers into a pure PEDOT:PSS, as can the hydrogel state). (**b**) Cryo-TEMM image of a pristine PEDOT:P SS (**i**) solution, (**ii**) 3D-printed conductive polymer ink, and (**iii**) 3D-printed conductive polymer after dry annealing. (**c**) and (**i**) Images of PEDOT:P SS nanofibril concentrations in re-dispersed suspensions and (**ii**) conducting polymer ink viscosity vs. PEDO T:PSS nanofibril concentration. (**d**) SEM images of 3D-printed polymer meshes. (**e**) Sequential images for the conducting polymer ink’s 3D printing of a 20-layered mesh structure [Red arrows display printing process in an alternate manner in each layer]. (**f**) and (**i**) Conductivity of 3D-printed dry (17 μm) and hydrogel (78 μm) conducting polymers vs. bending cycles. (**ii**) Nanoindentation of dry and wet 3D-printed conducting polymers. (**g**) and (**i**) 3D-printed soft neural probe images (**top**) and mouse with inserted probe (**bottom**). (**ii**) Soft neural probe 3D-printed with conducting polymer and PDMS inks. Three-dimensionally printed soft neural probe, magnified view. Reprinted with permission [119].

Recently, conducting polymers were also printed using 2PP. They may be used in power production, wearable electronics, and bioelectronics. Hyunwoo Yuk revealed a high-performance 3D-printable conductive polymer ink called poly(3,4-ethylene-dioxythiophene): polystyrene sulfonate (PEDOT: PSS) [119]. With multi-material 3D printing, insulating elastomers, for example, can be easily incorporated into microstructures made of conducting polymers, maintaining the high aspect ratio and the microstructure’s accuracy. Figure 7 illustrates the possibility of creating highly conductive and efficient hydrogel microstructures using 3DP conducting polymers. Due to their fluidity, conducting polymers cannot be used directly in 3D printing [120]. A PEDOT:PSS aqueous solution can be easily converted into printable ink, providing conducting polymers with the rheological characteristics needed for 3D printing (Figure 7a). Pure PEDOT:PSS solutions have low viscosity and show a sparse distribution of PEDOT:PSS nanofibrils (Figure 7(ai,aii)) (below 30 Pa s). Figure 7(ai) shows that highly concentrated PEDOT:PSS nanofibrils can make 3D-printable conductive polymer ink. This is comparable to how concentrated cellulose nanofiber solutions may be used to produce 3D-printed specimens. Isolating PEDOT:PSS nanofibrils via lyophilization of the pure PEDOT:PSS solution is the first step in testing this idea. Due to the delayed ice crystal formation during lyophilization at high temperatures, researchers lyophilized in a cryogenic environment to avoid the overproduction of PEDOT-rich crystalline domains among PEDOT:PSS nanofibrils that had been solidified in a liquid [121]. After being separated, the PEDOT:PSS nanofibrils were re-dispersed in a binary solvent combination of 85:15 *v*/*v* water:DMSO to produce saturated suspensions (Figure 7a(ii)). As the concentration increased, the suspensions gradually transformed from liquids to thixotropic 3D printable inks, as shown in the CryoTEM picture of pure PEDOT:PSS and nanofibrils entangled in the solvent, producing reversible physical networks in Figure 7b,c. The PEDOT caused this: the PSS nanofibrils created reversible physical networks via entanglements inside the solvent. Measurements of the conducting polymer inks’ rheology (Figure 7c) demonstrated the shift in 3D-printed inks from low-viscosity liquids (a low concentration of PEDOT:PSS nanofibrils) to physical gels (a high concentration of PEDOT:PSS nanofibrils). Conducting polymeric inks with low PEDOT:PSS nanofibril concentrations (1–4 wt%) cause the lateral spreading of inks during 3D printing on the substrate due to their low viscosity and negligible yield stress (Figure 7c(ii)).

However, as PEDOT:PSS nanofibril concentrations in conducting polymer inks rise above >8 wt%, the nanofibrils cluster into huge clumps that might clog printing nozzles. The intermediate range of PEDOT provides the ideal rheological characteristics and 3D printability with concentration (5–7 wt%). The rheological properties and printability of the conductive polymer ink used in 3D printing exhibit no significant alterations over a one-month storage period at an ambient temperature [96]. The outstanding printability of the conducting polymer ink opens the door for a broad range of cutting-edge 3DP application domains, such as creating high-resolution, high-aspect-ratio structures (Figure 7d). Researchers use nozzles of varied sizes (200 μm, 100 μm, 50 μm, and 30 μm) to print grid patterns of conductive polymer ink (7 wt% PEDOT:PSS nanofibril) to demonstrate microscale high-resolution printing (Figure 7e). Three-dimensional printing conducting materials retain strong electrical conductivity after 10,000 cycles. Conductivity reaches 100 S cm^−1^ in the dry state and over 15 S cm^−1^ in the hydrogel state, respectively (Figure 7(fi)). The mechanical characteristics of 3DP conducting polymers are measured utilizing nanoindentation measurements. The dry Young’s modulus (Y.M) values for 3D-printed conducting polymers are 1.5 ± 0.31 GPa, which is comparable to the values published for dry PEDOT:PSS40 (Figure 7(fii)). Hydrogel 3D-printed conducting polymers, on the other hand, have a Y.M of just 1.1 ± 0.36 MPa (Figure 7(fii)), making them mechanically equivalent to soft elastomers such as PDMS (Y.M: 1–10 MPa). Due to their softness and ability to gradually engage biomechanically with biological tissues, printed conducting polymer hydrogels may be beneficial in bioelectronic devices and implants. The 3D-printed delicate neurological sensor was put into the dorsal hippocampus (dHPC) of a mouse with the help of a plastic catheter (Figure 7(gi)). For two weeks, the soft neural probe made with 3D printing could reliably record the local field potential and other brain processes in the free mouse. A delicate neural sensor that can record bioelectronic signals in situ is easy to make.

Unlike conventional fabrication techniques such as electron beam lithography, which necessitate post-assemblies and intricate multi-step strategies, the high-resolution multi-material 3D printing capability enables researchers to print electrodes using conducting polymeric ink and the insulating encapsulation (PDMS-ink) of the neural probe with a straightforward, prolonged printing technique in less than 20 min (Figure 7(gii)). Thanks to its exceptional 3D printability and characteristics, conducting polymer ink printed by 3D printing holds promise as a simple, streamlined method for fabricating multi-material conducting polymer configurations and sensors with a high resolution. This research showed that 2PP may be used to fabricate 3D scaffolds from a CAD model in various ways. The high resolution of 2PP makes it possible to define both the scaffolds’ porosity and their microtopography simultaneously, which is impossible with any other method. According to the findings, the precursor (methacrylamide-modified gelatin) preserves its enzyme-mediated breakdown capacity following the polymerization process and may be cellularly sensitive. Furthermore, 2PP’s GelMOD-based scaffolds promote osteogenic lineage development and the adherence of porcine mesenchymal stem cells. Based on these findings, 2PP shows promise as a tool for creating photosensitive polymer scaffolds for use in TE.

In addition, polymer/semiconductor composites may be created utilizing polypropylene (PP). Several different nanoparticles are included in this combination, including TiO_2_, ZrO_2_, CdS and HA, and PbSe [122]. A piezoelectric barium titanate nanoparticle-containing material, such as Ormocomp, was used to print bioinspired 3D structures designed to look like the trabeculae of spongy bone [123]. This approach is also used to print novel ferrofluids with methacrylate-modified Fe_3_O_4_ nanoparticles at less than 3% concentration. These 2PP-printed micro-springs and micro-turbines demonstrated a magnetism response and motion control [124]. MPP and 2PP display the pixel-by-pixel control of printing characteristics at resolutions as low as a few hundred nanometers. The microstructures developed by Klein et al. were constructed using PEGDA/Irgacure 369 and PETTA [125]. After fibroblasts formed on the scaffolds, an extracellular matrix (ECM) protein adhered more to the Ormocomp component. Soft polymers have received less attention because of the frequent usage of acrylic and epoxy resins in MPP. Gold nanorods were incorporated into programmable layers by Moller et al. and then tested [126]. When combined with light stimulation, plasmonic heating and light morphing produced large-amplitude deformations, enabling reversible shape morphing and exhibiting many applications in sensors, artificial muscles, and switching systems. It is possible to circumvent the printing speed limitation of 2PP/MPP-based SLA systems via dynamic and projection-based monomer curing methods [127].

#### 3.2.4. DLP Printing

Photosensitive monomers and oligomers are used in both SLA and DLP polymerization, while DLP uses UV light to project an entire layer and cures much quicker than SLA [128]. The restricted variety of materials available with DLP and regular SLA is a disadvantage of both technologies. A common characteristic of free radical and cationic photoresponsive photocurable resins is that they are cured with high brittleness when cured in light. Griffin et al. created high-performance elastomeric materials to address the highly crosslinked framework and fragility of printed specimens. This research aimed to overcome the challenges associated with the highly crosslinked structure and fragility often seen in printed products [129]. PDMS composites were made by merging vinyl-terminated poly (dimethylsiloxane) polysiloxanes with different molecular weights. Modifying the amount of reinforcement fillers (fused and precipitated silica (such as 5% to 20% silica concentrations)), photoresists, and photoinitiators used in silicone/silica composites allowed the researchers to tailor the mechanical properties and durability of the composites to their specific needs [130]. Soft robotics and biomedical equipment might benefit from the use of materials that are highly malleable, biologically friendly, and cytotoxin-free. Preceramic polymer resins made from silicon might have uses in the ceramics industry. DLP’s fast silicone solidification allows porous, cellular, and multilayer lattice structures to be swiftly prototyped, as well as rapidly prototyping porous, cellular, and multilayer lattice structures.

Preceramic silicone and preceramic silicone mixed with alkali–silica photoresists that included alumina particles were used by Colombo et al. to develop improved mullite structures [131]. Highly acrylate polysiloxane produced a 31.8% ceramic output when pyrolyzed in the air, and the material was accessible to photo-cure after pyrolysis. Prior to curing, alumina was combined with phenoxyethanol and polysiloxane to make a suspension and then applied to the surface. Neither the scattering nor the absorption of light had any effect on the formation of the structure or the adhesions between the layers, unlike the total geometry, which decreased by 36% without affecting the geometry or the structure in any way. Their strong creep resistance and low electrical conductivity suit electronic and optical applications well. DLP-processable elastomers with mechanical stretchability, which are widely accessible and can be used on electronic substrates and packaging, may be advantageous for both applications. On the other hand, carbon or metallic nanoparticles enhance conductivity and associated properties. Gao et al. created a composite polymer matrix; two percent of CNT has a wide range of sensitivities (0.01 percent–60 percent) in DLP-cured polyurethane (0.01–60%) [132]. The material compatibility of FDM and DIW is superior to that of DLP-printed sensors, so the vast majority of general strain or chemical sensors manufactured via 3D printing employ them. Metallic nanoparticles, such as nanocarbons, have the potential to perform chemiresistivity-based sensing similarly. Silver nitrate was used to sensitize PEGDA photosensitive monomers before they were exposed to a digital light system with appropriate photoinitiators, as reported by Fantino et al. [133]. At 405 nm and 365 nm, reactive orange dye was utilized to prevent the light from exiting the targeted illumination zone, which regulated the width of the layer under investigation. Afterward, post-fabrication was carried out using ultraviolet (UV) firing. After the initial production, additional radicals were generated from Ag nanoparticles via reducing Ag ions with homogeneous dispersion and high conductance. Soft robotics, conductors, sensors, and actuators are just some of the devices produced using DLP.

Over time, the mechanical deterioration of biomedical polymers may be reduced by utilizing a polymer matrix incorporating ceramic composites or even pure ceramics in in vivo applications to reduce mechanical degradation. In contrast, complicated ceramic morphologies and hierarchies are difficult to fabricate using conventional manufacturing processes; as a result, 3D printing provides significant customization potential for ceramic structures. Zhu et al. investigated surface-modifying compounds to boost the loading capacity of calcium phosphate (CaP) by up to 60% [134]. In addition to high precision and an adjustable macro-pore structure, the 3D-printed CaP ceramics also displayed remarkable mechanical strength, as well as selective cell adhesion and bone growth once the processing settings were optimized. Shen et al. employed DLP to produce zirconia implant scaffolds containing 2–20% hydroxyapatite [135]. Powder technology, a defoamer, printing, cleaning, and ceramic sintering were used to prepare printing ink. Mechanical tests demonstrated a comparable strength to that seen in bone engineering. Following this, deposition and CaP degradation were seen in cell proliferation and differentiation experiments.

### 3.3. Material Jetting MJ

#### 3.3.1. Water-Based Jetting Processes: Inkjet Printing

Due to its compatibility with various printheads, non-contact properties, and direct scaling, inkjet printing is a promising multi-material method. The constraint on ink formulation, on the other hand, has proven to be the most significant drawback. To maintain the viscosity of the ink below 50 mPa s, it is required to regulate the particle amount tightly and the molecular weight of the polymer used [136]. Raising or decreasing the solute-to-solvent ratio, decreasing the size of polymers, and increasing or decreasing the additives’ concentration may all affect ink’s rheology [137]. Due to the low viscosity requirement, the particle concentration will be restricted, and the surface patterns will be thin and heterogeneous. As a result of removing organic compounds that were previously employed to speed up printing, improving the printing resolution and postprocessing speed may become more challenging. To better understand how ink behaves during printing, it is required to examine fluid rheology individually [138]. Multijet and polyjet printing, for example, produce shear rates in the range of 10–100 kHz, depending on the application [139]. The utilization of a torsional rheometer for viscosity measurement is feasible; nonetheless, its capability to attain elevated shear rates or frequencies is constrained. The genuine residence time of inkjet is in the millisecond range, but that of capillary rheometers is hundreds of times longer. However, capillary rheometers, such as inkjet, are often used in applications with short residence times [140]. Using high-rate cameras and recorders to capture dynamic surface tension and viscosity data straight from fluid might remove these concerns. Based on the assumption that Newtonian fluids behave in a “free-shape” mode, this calculation takes advantage of surface tension and viscosity to restore drop form and suppress oscillations while keeping the fluid steady [141]. It is possible to see the evolution of a droplet using the drop-oscillating method. One of the most desirable fluids for jetted inks is shear-thinning, which does not behave in a Newtonian manner. Ink viscosity, influenced by particle size and concentration, may impact the composites’ deposition speed, mechanical properties, and functional attributes. Ink viscosity may be lowered using hot print heads or reactive diluent additives, among other methods [142].

Nanomaterials comprising carbon nanoparticles, ceramic nanoparticles, or metals are required to regulate the viscous behavior of slurry. Particles with significant volumes or weight fractions cannot be deposited using inkjet technology because the nanoparticles may settle due to gravitational and density discrepancies among the nanoparticles, dispersion fluids, and the ink employed in the process [143]. It is also necessary to include the surface tension caused by the cohesive forces imposed on molecules at the liquid’s surface, which must be considered. For printing nozzles to function properly, the surface tension of the ink should be in the center of the spectrum, between high and low values. Pendant drop tests are quick and straightforward in measuring water’s surface tension accurately. A syringe needle is used to spread a drop of solution across the chamber during the experiment. The droplet’s morphological shift from spherical to elliptical happens concurrently with an increase in syringe capacity, owing to the synergistic interaction of gravitational and capillary forces. It is possible to calculate the equilibrium surface tension, as well as the dynamic surface tension, of a given solute-rich slurry by taking images and measurements of drop morphologies, among other things. H_2_O has a relatively low surface tension, but the solvents THF, DMF, DMSO, and thiodiglycol have very high surface tension [144]. The optimal range for ink surface tension is between 40 and 70 mJ/m^2^ [145]. After printing, annealing, curing, and sintering, the layers may eliminate holes, poor adhesion, discontinuities, fractures, and contaminants [146]. This technique can fuse particles, lowering their melting points and enabling them to be merged. Several technologies are necessary to construct high-resolution structures with outstanding performance. The effect of particle–polymer interaction on the inkjet printing of hydrogel composites for 3D bioprinting applications was investigated. Inkjet printing was used to produce PEG hydrogels containing SiO_2_ nanoparticles. The researchers discovered that the interfacial adhesion between the particles and the polymer matrix was essential for the structural stability of the printed composites [147]. Padmavathi discovered that printing factors such as nozzle diameter and printing speed affected the composites’ interfacial adhesion and ultimate mechanical properties [148].

Researchers looked into the impact of particle–polymer interactions on the inkjet printing of calcium phosphate (CaP) scaffolds for bone tissue engineering applications [149]. The scaffolds were printed using inkjet printing and a mixture of CaP particles and polycaprolactone (PCL) polymer [150]. The interfacial adhesion between the CaP particles and the PCL matrix was discovered to be essential for the mechanical properties and biocompatibility of the printed scaffolds [151]. Printing factors such as nozzle diameter and ink composition affected particle–polymer interactions and the final properties of the scaffolds. The impact of particle–polymer interactions on the inkjet printing process of hydrogel scaffolds was studied when loaded with different kinds of nanoparticles, such as SiO_2_, HA, and Fe_2_O_3_ [152]. The scaffolds were printed using a custom-built inkjet printing method, and their mechanical properties and biocompatibility were assessed. Cheng found that particle–polymer interactions affected the printed scaffolds’ printing resolution, mechanical properties, and biocompatibility [153]. The ink composition, printing parameters, and particle size and distribution all impacted the particle–polymer interactions and the end properties of the printed scaffolds. Magdalena looked at how different particle types, such as calcium phosphate and silica nanoparticles, interacted with alginate-based hydrogels during inkjet printing. Hydrogels were tested for their mechanical characteristics, swelling behavior, and degradation rate utilizing an inkjet printing evaluation system [153]. They discovered that particle–polymer interactions affected the printed hydrogels’ printing resolution, mechanical characteristics, and degradation rate. Besides this, the particle–polymer interactions and final characteristics of the printed hydrogels were affected by the ink composition, printing settings, particle size, and concentration.

**Figure 8:**
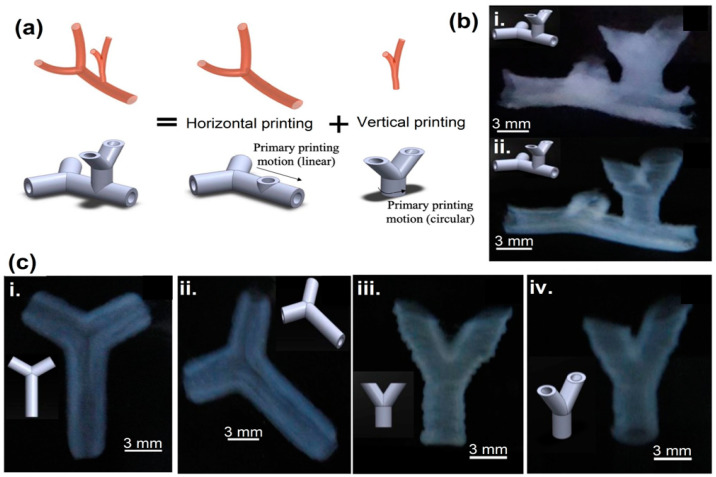
(**a**) Basic tubular configuration of blood vessel network. (**b**) Vertical–horizontal bifurcations. (**i**) The cellular structure incorporates horizontal and vertical bifurcations and is fabricated using inkjet printing technology, (**ii**) The accompanying insets depict the designed structure in detail. The corresponding structure composed solely of alginate exhibits greater geometric accuracy. (**c**) Front and global overview of bifurcated alginate configuration printed (**i**,**ii**) horizontally and (**iii**,**iv**) vertically. Reprinted with permission [156].

Arcs using spheroids or bioinks as building blocks were also used in organ printing, which has the ability to create freeform, layer-by-layer, 3D living organs. The difficulty in appropriately vascularizing the tissues is frequently highlighted as a barrier to developing 3D organs [154]. Thus, the development of 3D biological vascular trees is critical to the viability of the proposed organ manufacturing methodology. Crosslinking agents and supports are used to create vascular-like cell structures via inkjet printing incorporating a calcium chloride solution [155]. Using a buoyant force, this method allows the printing of freeform features that span and hang over the edge. It is necessary to correct horizontal tubular systems’ axially varying deformation to maintain a constant axial diameter. Sodium alginate and mouse-fibroblast-based alginate bioinks were used to print vascular-like structures comprising transverse and longitudinal branching. Even after 24-h of incubation, the printed cellular tubes’ fibroblast cell viability after printing was determined to be greater than 90% when the control effect was taken into account, as shown in Figure 8 [156].

#### 3.3.2. Multijet and Polyjet

Multijet and polyjet are capable of various applications because of their electrohydrodynamic (EHD)-based printing technology [157]. The capillary force of the tiny printing needle must be countered by smaller printing features, which require the reduction of printing channels and an increase in printing pressure. Printer head clogging is a natural consequence that occurs on a regular basis. To avoid the possibility of droplet materials spilling and causing thickness irregularity or spatial heterogeneity, a smaller printing head may not be able to improve the printing resolution [158]. While traditional inkjet systems employ acoustic or thermal stimuli to push liquids out of the jetting channel, the EHD jet uses an electrical field to force liquids out of the channel. The conducting needle can only reach the ink if back pressure is applied to the supply. The Taylor cone is created when an electrical voltage is supplied to a meniscus and the meniscus deforms [159]. Droplet formation will be accelerated beyond surface tension limits due to the electrical stresses induced by the electric field in the surrounding environment.

The presence of ions in the composition of inks leads to the formation of droplets or streams of droplets. Following a set of broad guidelines, the EHD jet should be used. As a starting point, it is recommended that the diameter of the EHD printhead be limited to a minimum using numerous particles, comprising organic, inorganic, or metallic materials. However, the needle must be larger than the particle. To make things even more complicated, the electrical field intensity can be adjusted to account for the increase in droplets’ deposit width when the electrical field strength is raised. For example, in the 700–1000 V range, the printing line width might vary from 1 to 10 m. Ultimately, the morphology (coiled or continuous) of the printing line, as well as the diameter of the fiber, may be impacted by the stage movement speeds of the EHD platform’s stage movement. EHD printing has a few limitations [160]. The stability of electricity, as well as its importance to the printing resolution, are uncertain. The printing resolution can be impacted by electric field strength vibrations, platform movement, and electrostatic resistance from printed layers, particularly when layers are deposited in the print’s height direction. When it comes to printing silver nanowires with high-resolution paths, Lim et al. used an acrylic polymer/silica nanomaterial composite to cover the silver route, completely eradicating silver’s presence while simultaneously improving mechanical characteristics and durability [161]. The use of EHD to print PSS/CNT composites revealed the importance of solvents, voltages, printing speeds, and substrates in the process of optimizing for electrode applications, which was previously unknown [162]. According to Chang, EHD-printed and stretched fibers containing PEO and PCL cores were merged to form a unique structure with increased elasticity and restoration [163].

#### 3.3.3. Binder Jetting Printing

Binder jetting is a process in which liquid binders are jetted onto scattered powders using an inkjet printer head. If the binder exhibits the appropriate characteristics regarding rheology, wetting ability, and stability, it can be considered highly favorable. Tiny picoliter droplets of binder ink are dropped onto a polymer powder [164]. 

Higher-impact velocities provide bigger impact radii and, as a consequence, worse spatial resolution. It is necessary to maintain an equilibrium between droplet dispersion and infiltration depth to keep droplet distribution and infiltration depth in balance. Surface tension and capillary processes are responsible for this. Current attempts have focused on powder densification using heating or pressure to homogenize the powder via binder addition [165]. Shen X and colleagues conducted a study in which they tested graphene inks and examined the properties of PVA/graphene composites using binder jets. They tested them for evaporation, capillary suction, sorption, and aggregate deposition, evaluating their flexibility and enhanced conductivity [166]. In another study conducted by Liravi et al., it was found that the creation of high-performance electrodes could be achieved by jetting palladium dispersion into graphene oxide powder. The gravimetric and areal capacities of graphene layers with pallium insertion are superior [167]. Developing composites with flexibility and conductivity via ink stability was the key to making wearable devices more flexible and super-capacitors more conductive. If the nozzle is not cleaned regularly, it will get clogged. Liu et al. investigated a silk/PVA blend using water-based solvents, yielding positive outcomes [168]. In this study, the flowability and spreadability of a 20-micron silk powder were better than those of a 5-micron silk powder. To achieve an optimal 200 nm resolution and superior composite dynamics, the silk/PVA ratios and printing parameters were tweaked. It is now possible to manufacture water-stable printed components using a glutaraldehyde infusion and emersion post-crosslinking, expanding the composite’s potential uses in biomedicine. To fuse metallic materials similarly, a solution or gel-based ink may be used to prevent the printing nozzle from being clogged frequently. This may be accomplished by employing metal-free inks (for example, metallic organic decomposition inks) to improve flowability [169]. Increasing the solubility of metal salts was one way in which Bai et al. improved the ink’s rheology using Cu as ligands [170].

The bonding strength of polymer binders varies, depending on their composition. Wei et al. explored the effect of three binders (PVP, PAM, and PVA) on a binder-jetted bioceramic [171]. The influence of the polymeric matrix on the cohesion energy density, bond strength behavior, and morphological properties was demonstrated using molecular dynamics simulations. Thermal treatment was required after printing to remove pores and enhance the linkages between the print particles. The inability to monitor or foresee the annealing contraction of printed components is a key disadvantage of this approach [172]. Zhou et al. employed the utilization of polycaprolactone (PCL) and calcium sulfide (CaS) structures through the binder jetting method. Upon the infusion of the PCL compound into the inter-particle void, a notable enhancement in the mechanical characteristics of the composite material was observed [173]. PCL materials provided structural support for the scaffold to resorb, allowing aid from the PCL coating. Powder technology, binder composition, and postprocessing dynamics are a few issues that will require future investigation. In comparison to SLS, binder jetting has several advantages, including its high compatibility with a greater variety of materials, (ii) its room-temperature fusion, which prevents polymer oxidation or degradation, (iv) and the absence of support structures, which are required in FDM, and, (iv) being able to alter material density through the use of void coalescence and temperature tuning., it is theoretically conceivable to print on almost any polymer or composite powder using this method [174]. It is feasible to disperse binders of different colors using several printheads; infiltration or heat treatment is required after printing in certain areas since they are so porous.

### 3.4. Direct Energy Deposition (DED)

The DED 3D printing technique makes components by directly melting materials and depositing them layer by layer on the workpiece. This additive manufacturing method is often employed with metal powders or wire source materials. DED includes electron beam additive manufacturing (EBAM) and laser deposition welding (LDW). 

#### Electron Beam Melting 3D Printing

**Figure 9:**
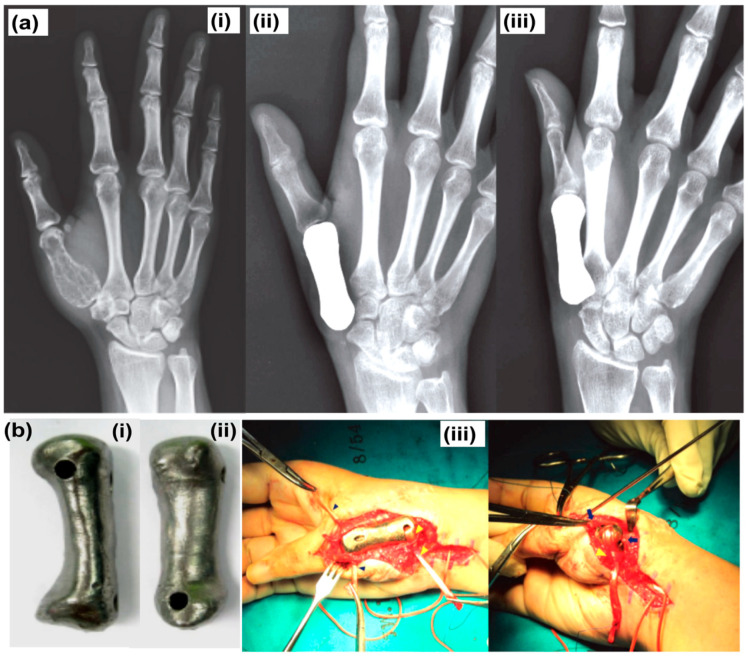
(**a**) (**i**) Radiograph demonstrates an expansile osteolytic lesion extending the first metacarpal’s full length. (**ii**,**iii**) Patient-matched first metacarpal prosthesis radiographs. (**b**) Before-implant-prosthesis images: (**i**) anterior aspect (**ii**) and volar aspect (**iii**). Intraoperative images showing 3D-printed titanium first metacarpal prosthesis with ligament reconstruction in proximal and distal portions: free palmaris longus tendon graft (blue arrow), flexor carpi radialis tendon (yellow arrow), and extensor pollicis brevis tendon (blue arrow). Reprinted with permission [175].

A 37-year-old woman contacted a clinic after four months of severe edema and limited thumb movement. X-rays revealed a massive osteolytic lesion that ran the length of the first metacarpal. An MRI scan revealed the tumor’s atypical growth, which had caused it to invade the cortex. The tumor had infiltrated the surrounding soft tissue and wrapped itself around the first carpometacarpal joint, as shown in Figure 9(ai).

The patient reported no pain or tumor recurrence at the latest follow-up, which occurred 2 years after surgery. The thumb was found to have shrunk by 5 mm. Clinically and radiologically, the MCP and CMC joints were stable (Figure 9(aii,aiii)). Resection was done via en bloc. As a stopgap measure, bone cement was used to bridge the defect after removing the first metacarpal and trapezium. After waiting six months, a second MRI showed no signs of the tumor’s return. After a conversation with the medical team, the patient ruled out the use of autogenous bone grafts in the ongoing reconstructive plans. Afterward, surgical intervention with a bespoke prosthesis was made available to the patient. The unique mold was 3D-printed via the electron beam melting process using a CT scan of the patient’s left metacarpal as a mirror image. The remaining titanium prosthesis was produced from this mold. The proximal and distal ends of the prosthesis featured several holes intended for ligament repair and provisional attachment (Figure 9(bi,bii)). A cement spacer was removed from its biomembrane and enclosed via a longitudinal incision. After the area was prepared for the titanium prosthesis, the cement spacer was removed after the ligament repair (Figure 9(biii)).

### 3.5. Extrusion-Based 3D Printing

Extrusion-based AM develops 3D structures by accumulating layers of material on a substrate. Generally, fused deposition modeling (FDM) and LDM techniques are included in this category.

#### 3.5.1. Fused Deposition Modeling (FDM)

The FDM technique is compatible with various materials, including the polymers and particles most typically employed in conventional manufacturing processes. According to its intended usage, material for FDM may be classified as a commodity, an engineering type, or a high-performance type. Amorphous polymers or semicrystalline resins (e.g., PMMA ABS, PS, and PVC) are cheap plastics for FDM (such as PP, PE, and PEVA). PC, PVA, and other amorphous and semicrystalline polymers are included in this group (e.g., PLA, PLGA, PA, PCL PBT, and PET) [176]. Some high-performance resins have melting and degrading temperatures that are close to one another. (e.g., the PAEK family). Amorphous and crystalline polymers may be combined in a single filament via the use of polymer blends. As a result, items produced with FDM may have enhanced mechanical characteristics while experiencing less shrinkage and interlayer debonding. On the other hand, the miscibility of these filament mixes poses a significant barrier in their fabrication. Compatibilizers, which are valuable additives to prevent phase separation between component polymers in filaments, are becoming more popular [177]. Unfortunately, because of limitations in the filament format, the range of materials that may be used with FDM is restricted. The majority of filaments now available come from extrusion-based manufacturers. The investigation of additional polymer powders in extruded filaments has the potential to broaden the range of FDM materials available. Many FDM-printed materials may be combined and extruded via several custom-designed nozzles [178]. Three methods for printing particle-included polymer composites are sintering, extrusion, and direct printing. The integration of a polymer solution and in situ gelation before extrusion (for example, frontal polymerization), the fusion of liquid state epoxy resins on moving continuous fibers (e.g., carbon fibers) with core-shell fibers, and the extruding of pre-impregnated filler particles with polymer nanocomposites are examples of advanced extrusion techniques. Continuous fibers, as a reinforcement in composites with mechanical, thermal, and electrical characteristics, are superior to pure polymers. Unlike single-filament manufacturing, two independent material supplies are available for printing the polymer and reinforcing the fiber when using a twin-extruder FDM printer [178]. FDM-produced materials are criticized for their weak mechanical durability in compression, tension, and flexion testing. The material mechanics of FDM are influenced by the limited range of polymers that may be used, the existence of voids, poor accuracy in layer width, the anisotropic behavior of imprinted patterns, and the interfacial adhesion instability between molten polymer layers, all of which are factors to consider [179]. One of the most significant shortcomings of FDM is its poor interlayer bonding strength [180].

Interest in bioengineered artificial blood vessels has increased during the last ten years. Due to the existing lack of viable surgical alternatives, smaller vessel diameters are of special interest. Cells must be suspended in specific hydrogels for bioprinting to function. In a degradation process using MAA anhydride, the lysine and hydroxyl residues of gelatin were replaced with methacrylamide and methacrylate side groups, named GelMA [181]. Due to their adjustable physicochemical qualities and beneficial clinical manifestations, GelMA hydrogels are frequently utilized in TE and stem cell therapy. Arginine–glycine–aspartic acid (RGD) sequences that promote cell adhesion and matrix metallorproteinase (MMP)-sensitive peptide motifs that permit cell growth and dissemination within the scaffolds are just two examples of the many peptide patterns found in GelMA that are strikingly similar to the fundamental elements of a native extracellular matrix (ECM). In addition, when subjected to UV light with a photoinitiator, GelMA may form a covalently crosslinked hydrogel; it has also been utilized in extrusion bioprinting [182]. Utilizing a 3D micro-extrusion bioprinter, Lei Xu and coworkers planned to print a blood-vessel-like heterogeneous bilayer with a tiny diameter using gelatin methacryloyl (GelMA) bioink in a single cycle [183]. The integration of hyaluronic acid (HA), glycerol, and gelatin into GelMA led to advancements in printability, durability, and biocompatibility, therefore enabling the fabrication of a bioink. To create a heterogeneous bilayer, two different GelMA bioink concentrations were used, each with its distinct pore size. The study utilized a GelMA bioink with a significant concentration, comprising 6% GelMA, 2% gelatin, 0.3% HA, and 10% glycerol. The use of human umbilical vein endothelial cells (HUVECs) embedded in bioink was employed to stimulate the development of endothelial linings inside the human body.

**Figure 10:**
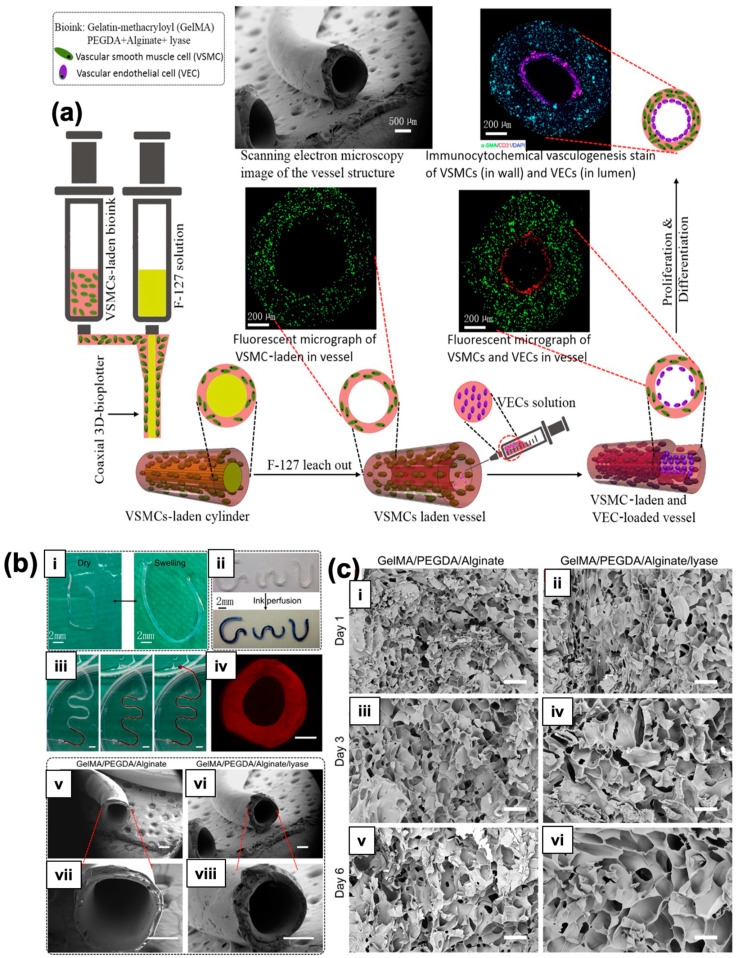
(**a**) Illustration of 3D bioprinting displaying biomimetic two-layer blood vessel. (**b**) 3D-bioprinted biomimetic blood vessels. (**i**) Printed vessel remained intact (swelling and dry status). Vessels perfused with (**ii**) blue and (**iii**) red ink fluid. Scale bar = 5 mm. (**iv**) Cross-sectional view of vessel structure. Scale bar = 300 μm. SEM images of the blood vessel structure (**v**,**vi**) without and (**vii**,**viii**) with a lyase composition. Scale bar = 500 μm. (**c**) SEM images of cross section of bio-printed blood vessels (**i**–**iv**) without and (**v**,**vi**) with lyase. Scale bar = 100 μm. Reprinted with permission [186].

A reduced concentration of GelMA bioink was employed to incorporate smooth muscle cells (SMCs) and produce an extracellular matrix consisting of muscle fibers. Mechanical aspects, such as suturability, Instron mechanical testing, and biological properties, such as viability, proliferation, histological characterization, and the effectiveness of bioprinted implants resembling blood vessels, were evaluated. The resulting heterogeneous-bilayer blood-vessel-like structure is 20 mm in length, has a 4.0 mm lumen, and maintains high cell viability and proliferation levels. Their findings provide a fresh approach to the biofabrication of blood vessels with a narrow internal diameter. A group led by Lei Xu employed GelMA bioink in varying concentrations and pore sizes to produce a heterogeneous bilayer. The variation in cell sizes necessitated the presence of pores with different diameters in scaffolds to promote optimal cell growth, migration, and differentiation. Different formulations of GelMA bioink were studied to see how they affected pore size distribution (from 3% to 15% GelMA by weight/volume). The results showed that, when GelMA content in bioink increased, the pore diameters in the structures decreased. The bioink’s pore size was unaffected by the addition of gelatin, glycerol, and HA. The printability of bioink can be roughly estimated by its viscosity. Greater printability is a possibility due to increased viscosity. They illustrated that the storage modulus in the three types of printed structures was greater than the loss modulus after crosslinking. This structure contains an inner layer that is printed with ink containing HUVECs. As a circular, 6% GelMA bioink was applied, and an outer layer printed with SMC-loaded 4% GelMA bioink was used as two concentric rings. After the layers were crosslinked, the blood vessel measured 4 mm in diameter, 0.8 mm in thickness, and 20 mm in height. Stitching together two sliced tubular structures confirmed their suturability. The tensile strength was tested using the Instron Mechanics Tester on day 1 of maturation. The structures comprised 4% and 6% GelMA bioink, and a combination of 4 and 6%. The GelMA bioinks all had similar stress–strain profiles. Hence, an upsurge in bioengineered artificial blood vessels was observed. The surgical options for vessels with a diameter of <6 mm are particularly interesting [184]. The fabrication of artificial organs and tissues now has more options due to 3D bioprinting. The modulable pore size and ultraviolet (UV) curable properties of GelMA make it a desirable hydrogel for tissue engineering applications. Similarly, synthetic vascular grafts have been marketed for clinical use; they are now limited to large-diameter arteries (>6 mm). However, small-diameter vascular (<6 mm) replacements continue to pose considerable clinical issues across the globe [185]. Xuan Zhou and his colleagues used an advanced coaxial 3D-bioplotter platform to generate innovative, programmable, small-diameter blood vasculature with two separate biomimetic cell layers (vascular endothelial cell (VEC) and vascular smooth muscle cell (VSMC)), as shown in Figure 10 [186].

To imitate the normal makeup of the blood vessel, VSMCs were loaded in the vessel wall, and VECs proliferated in the lumen. A unique bioink was developed using VSMCs loaded with gelatin methacryloyl (GelMA)/polyethylene(glycol)diacrylate/alginate and lyase (Figure 10a). This particular design promotes nutrition exchange in an ambient environment while also improving loaded cell proliferation in the matrix pore without the space constraints associated with drug encapsulation. The loaded VSMCs developed slowly in the vessel wall as the alginate was progressively dissolved by lyase, allowing additional room for cell growth in matrices. The vessel displayed considerably perfusable and mechanical qualities at varied flow velocities, flow viscosities, and temperature conditions using computational fluid dynamics modeling. Furthermore, both VSMCs in the scaffold matrix and VECs in the lumen proliferated gradually over time, resulting in a considerable two-cell-layered structure (Figure 10b). Cell proliferation was visually validated by staining the vessel walls and lumen with the markers of alphasmooth muscle actin and cluster of differentiation, which are both associated with angiogenesis. Furthermore, the findings were quantitatively validated via gene analysis, indicating strong angiogenesis expression in the blood vessels.

#### 3.5.2. Low-Density Polyethylene Printing (LDM)

In addition to direct-write fabrication, 3D dispersion, 3D plotting, robot 3D extrusion, and robot-assisted shape deposition, direct-write fabrication, commonly known as LDM/DIW, is a method that utilizes a pressurized syringe to print layers of material. Depending on the application, the material layers may comprise a gel, slurry, or solvent suspension. LDM polymer printing in biological domains is the most prevalent among several applications [187]. There are specific needs for biomedical applications. Bioprinting offers a unique advantage over traditional manufacturing by enabling compliance with stringent standards that are typically challenging to meet. Bioinks made from natural polymers, fibers, and molecules make it possible to print medicinal products, tissues, and organ systems through 3DP. A significant obstacle encountered in the domain of bioprinting is the limited availability of suitable materials within the biomedical sector. The materials used should possess the dual qualities of being biocompatible and biodegradable in the final product environment. Due to differences in healing effects within the human body, as well as physical disparities across patients, certain individual organs may need tailored replacement. Pain, mesh erosion, and infection are all risks associated with the sole commercial meshes of polypropylene scaffolds used exclusively for this purpose. Choosing a more natural biodegradable composite material minimizes damage and encourages successful regeneration [188]. When external inputs are used in conjunction with the printing process, folding, rolling, movement, shrinkage, expansion, and assembly may be included in the printing process, resulting in 4D printing with various applications in soft robotics and other application fields [189]. Temperature fluctuations, electricity, light, wetness or liquid immersion, pH, and other parameters may be used as stimuli. In addition to complex processing (such as etching and deposition), traditional semiconductor or photonic industries frequently employ patterning technology (such as polymer assembly) [190]. In the context of 3D printing, bilayer or multilayer heterogeneous designs may easily produce mismatched stresses, resulting in 3D structures that self-fold or self-roll in reaction to changes in their surrounding environments. The programming activity of the form memory fibers can potentially produce temporal dependency in the configuration of the shape memory fibers, depending on the programming activity (e.g., 4D printing) [191].

It is possible to build 3D configurations using printed laminates in thin-plate forms, such as bent, coiled, and twisted strips folded forms, and complicated contoured geometries with nonuniform, spatially variable curvature. Three-dimensional printing composites may be performed with the least work if a CAD file detailing the fiber placements is used in conjunction with a printer [192]. The achievement of anisotropic thermomechanical behavior may be accomplished via various modifications to the filler architecture. These modifications include alterations to the form, size, orientation, and spatial variation of the filler particles. It is possible to convert composites between their fundamental flat rectangular strip and their converted phases or forms, including folding, wrinkling, and helices, among other shapes, using simple heating and stretching procedures, chilling and cooling, and releasing processes. The Qi group also worked on a mathematical model with intelligent, active hinges, which was utilized to create origami folding patterns using the material system [193]. This model shows how a flat polymer sheet assembly may be folded into a pyramid or an origami airplane using a simple folding technique. Fiber size, hinge length, stresses, and temperature are all aspects to consider when designing a flat polymer sheet assembly. These self-folding structures may be used for various applications, including flexible electronics, robotics, solar cell packing, space building, and deployable biomedical devices; they may also save money by reducing the need for expensive infrastructure (e.g., robotic arms). Shape memory polymers with a wide glass transition temperature range (0 to 100 °C) are used to achieve the desired elastic qualities. Generally speaking, crosslinks can be divided into two categories, chemical or physical crosslinks, which are used to create the permanent 3D structure and transition temperatures (Ttrans, which can be either glass-transition or melting-point), which are used to regulate the molecular switching segments and secure the temporary shape [194]. The material can become temporarily deformed when the temperature is elevated over its transition point for molecular switching. Hydrogels are frequently utilized in both 3D printing and 4D printing processes. The Tasar group reported on the usage of a poly (N, N-dimethyl acrylamide) matrix, as well as stimuli-responsive poly (N-isopropyl-acrylamide) polymers implanted at various levels with clay particles and cellulose fibers embedded at various layers [195]. Stiff fillers were placed in several orientations at different periods, allowing them to control swelling and elastic anisotropies.

Phosphorus-sensitive 3D-printed polymers and composites constitute a new subgroup of intelligent materials capable of switching from one structure to another when subjected to changes in pH greater than or equal to a threshold pH value [196]. In polymer matrices, either polyacid or polybasic in nature, ionizable functional groups that undergo protonation and deprotonation in response to pH are present. Due to electrostatic repulsion between the polymer chains when they are in the charged state, they stretch into a coil shape, which subsequently collapses back into a globule shape when the charges on their functional groups are neutralized [197]. Connal et al. reported on the 3D printing of P2VP/ABS composites for pH-responsive flow regulators using a stereolithography process [198]. The P2VP composition expands and shrinks in response to the pH of the solution, resulting in dynamic and reversible shape modifications. FDM-printed poly (methacrylic acid/co-ethyl acrylate) with core-shell structures, which demonstrated structural integrity at acidic pH and a controlled drug release at neutral physiological conditions, included (i) LDP-printed collagen, which was biocompatible and responsive to biomedical conditions [199], and (ii) SLA-printed keratin, a polymer derived from human hair, which demonstrated structural integrity at acidic pH and controlled drug release [200].

### 3.6. Visible-Light-Mediated Printing

One potential drawback of using ultraviolet light for the photopolymerization of massive structures is that it has a shallow penetration depth. In the past decade, 1-[4-(2-hydroxyethyl)-phenyl]-2-hydroxy-2-methyl-1-propane-1-one (Irgacure 2959) and UV radiation (320–365 nm) have been the most-often-recorded technique for crosslinking GelMA [201]. UV light, however, has been found to affect cellular chromosomal and genetic instability. Tissue engineering could benefit significantly from using alternative photoinitiators that absorb the visible light (Vis) spectrum. Several other visible light photoinitiating systems, such as camphorquinone, eosin Y fluorescein, lithium phenyl-2,4,6-trimethylbenzoylphosphinate (LAP), riboflavin, and rose bengal, have been studied to create cell-laden hydrogels [202]. Only eosin Y and LAP have been used with GelMA so far [203]. In addition, these initiators have drawbacks, such as low water solubility, a complicated synthesis route, low photoreactivity, and cytotoxicity [204]. Moreover, using the phosphinate initiator for polymerization at a wavelength of 405 nm, which falls within the visible light spectrum, is accomplished by employing low concentrations of the initiator and light intensities by Fairbanks, B.D. This approach is not feasible for polymerizations initiated with I2959 due to its absorbance profile. Upon evaluation 24 h post-encapsulation, it was observed that the survival rates of human neonatal fibroblasts encapsulated in hydrogels polymerized with the phosphinate initiator were greater than 95%. This finding provides evidence of the cytocompatibility of the aforementioned initiating system [205]. A new light-triggered radical polymerization method involving water-soluble photoinitiators, ruthenium (Ru), and sodium persulfate (SPS) compounds, capable of absorbing photons in the visible light range, has recently been deployed by Lim KS et al. [206]. How to replace or heal diseased or damaged tissues using tissue engineering and regenerative medicine (TERM) techniques that combine cells in tissue engineering scaffolds has been extensively studied [207]. These techniques have been used to manufacture a variety of tissues, including bone, skin, and cartilage. The necessity for personalization, when individual patients require specialized tailored engineered tissue constructs depending on the size and shape of the targeted tissue defect, is currently a critical unmet problem in TERM. To manufacture patient-specific tissue constructions, various research groups have investigated combining cutting-edge biofabrication or bioprinting technologies with TERM, in which materials or cells are deposited layer by layer from patients’ three-dimensional (3D) imaging data.

Hence, visible light is beneficial for medical applications since it lacks UV’s damaging effects, especially in 3D bioprinting [208]. Oxygen inhibition immediately affects the print fidelity of 3D biofabricated and photopolymerized hydrogel constructions. Due to defective crosslinking, it frequently causes the unwanted physical collapse of manufactured constructions, a problem that typically goes unnoticed. Lim KS and his colleagues’ study compared a novel visible light initiating system, Vis + ruthenium (Ru)/sodium persulfate (SPS), to a more widely used ultraviolet (UV) + Irgacure 2959 system to describe a systematic approach to minimizing oxygen inhibition in photopolymerized gelatin methacryloyl (GelMA)-based hydrogel constructs [209]. Both systems reduced oxygen inhibition by raising the photoinitiator concentration and light irradiation intensity. However, at both high I2959 concentrations and UV light irradiation intensities, the UV + I2959 system proved harmful to cells. A further indication of the Vis + Ru/SPS system’s potential to biofabricate cell-laden constructs with high shape fidelity, cell viability, and metabolic activity is the fact that it produced better cell cytocompatibility. The encapsulated cells remained >85% viable even at high Ru/SPS concentrations and visible light irradiation intensities for up to 21 days.

Besides this, 3DP methods based on lithography enable a higher spatial resolution than traditional extrusion-based bioprinting techniques and better simulating of the intricate architecture of biological tissues [210]. Additionally, freeform lattice and patterned structures can be created using lithographic printing via digital light processing (DLP), which is not possible with conventional 3D printing techniques [211]. The development of cytocompatible bio-resins and their use in lithography-based biofabrication have received less attention than the development of cell-laden bioinks for extrusion-based bioprinting, limiting the development of this promising technology. Malda and his coworkers fabricated a novel bio-resin based on methacrylated poly(vinyl alcohol) (PVA-MA), gelatin methacryloyl (GelMA), and a visible light photoinitiator based on transition metals [212]. The bioprinting of constructs with high-resolution features, in the range of 25–50 μm, was made possible using a visible light photoinitiating system, demonstrating high molar absorptivity. Resin biofunctionalization with 1 weight percent GelMA facilitated the adhesion and dissemination of endothelial cells planted on the printed hydrogels and the long-term survival (>90%) of encapsulated cells for up to 21 days. The successful bioprinting of cell-laden hydrogel constructions with a high-resolution, intricate, and ordered architecture was accomplished, and the encapsulated cells maintained their viability, uniformity, and functionality. Encapsulated stem cells demonstrated bone and cartilage tissue formation, highlighting the promise of these DLP-bio-printed hydrogels for tissue engineering and biofabrication [213]. Hence, the PVA-MA/GelMA bio-resin is a promising material for biofabrication and offers significant pointers for advancing the lithography-based bioprinting of complex, freeform living tissue mimics [214].

Similarly, Mubarak W. described how sugar beet pectin (SBP) was hydrogelized using visible light-mediated photocrosslinking and how it was used in extrusion-based 3D bioprinting [215]. Applying 405 nm of visible light to an SBP solution while it contained sodium persulfate (SPS) and tris(bipyridine)ruthenium(II) chloride ([Ru(bpy)_3_]^2+^) led to rapid hydrogelation (15 s). SBP, [Ru(bpy)_3_]^2+^, and SPS concentrations, together with the duration of visible light irradiation, could be adjusted to alter the hydrogel’s mechanical properties. Extruding inks comprising 3.0 wt% of SBP, 1.0 mM of [Ru(bpy)_3_]^2+^, and 1.0 mM of SPS allowed high-fidelity 3D hydrogel structures to be created. After 14 days in culture, human hepatoblastoma (HepG2) cells embedded in SBP hydrogels were still alive and metabolically active. Guo X.’s study shows that the 3D bioprinting of cell-laden structures for tissue engineering applications is feasible when SBP and a visible-light-mediated photocrosslinking technology are used [216].

Thus, the biofabrication phenomenon helps to precisely control the deposition of cells and biomaterials with the help of a computer and has demonstrated promising results in creating structures with complicated and organized designs that can mimic the organization of the original tissue [217]. Some biofabrication methods, such as extrusion printing and microvalve printing, show incredible promise over others for making large structures with greater therapeutic utility in tissue engineering. Laser-assisted printing, inkjet printing, and extrusion printing are also examples of biofabrication methods [218]. Among the many available hydrogel materials, gelatin hydrogels have demonstrated exceptional promise as bioinks for the biofabrication of organs and tissues such as the liver, skin, cancer models, and cartilage. Gelatin, a byproduct of collagen hydrolyzation, is very desirable as a target hydrogel or bioink for tissue engineering procedures because it both is water-soluble and contains numerous peptide sequences, such as RGD, that are known to stimulate cell adhesion and proliferation. Functionalizing gelatin with methacryloyl groups (GelMA) is a widespread practice in biofabrication; this allows for the development of irreversible covalent crosslinks after printing through light-activated radical polymerization [219]. Light curing is commonly used to permanently fix the shape of these 3D plotted/extruded GelMA hydrogel constructions containing photoinitiators. The photoinitiators crosslink the polymers by absorbing photons and dissociating them into radicals, which then propagate via the methacryloyl groups to form covalent kinetic chains.

### 3.7. 3D Bioprinting

The practical application of 3D bioprinting has garnered significant attention due to its ability to create tissues. The ability to precisely position and bioprint small tissue blocks, commonly known as spheroids, in three dimensions has emerged as a significant challenge within this field. Ayan B introduced a groundbreaking technique known as aspiration-assisted bioprinting (AAB) [220]. The proposed technique leverages aspiration forces to selectively isolate specific biologics and subsequently bioprint them in a three-dimensional format. The AAB platform offers support for various biofabrication techniques, such as scaffold-based or scaffold-free bioprinting, in conjunction with microvalve bioprinting. This method demonstrates a significant level of accuracy in positioning the bioprinted components, achieving a precision of approximately 11% relative to the size of the spheroids. Ayan B aimed to investigate the fundamental physical mechanisms underlying AAB (aspiration and bioprinting). The objective of the study was to gain a deeper understanding of the interactions between aspirated viscoelastic spheroids and the governing physical forces involved in the processes of aspiration and bioprinting. The field of bioprinting has demonstrated significant achievements in the fabrication of diverse biologics, encompassing a broad spectrum of dimensions. The components observed in the study encompassed discrete cells, specifically electrolytes, measuring approximately 400 μm in size. Additionally, tissue spheroids were identified, exhibiting a size range spanning from 80 to 600 μm. Furthermore, elongated tissue strands were observed, extending up to 800 m in length. Two applications were highlighted to demonstrate the versatility of this method: the precise organization of angiogenic sprouting spheroids and the spontaneous formation of osteogenic spheroids [220]. Besides this, Multicellular spheroids (MCS) have been used to create scaffold-free tubular tissue using a unique method based on a “Bio-3D printer”. Due to the scarcity of endothelial cells in synthetic vascular prostheses, few small-caliber vascular prostheses are currently therapeutically accessible. These endothelial cells are critical in controlling platelet activation, leukocyte adhesion, thrombosis, and vasomotor tone by generating vasoactive compounds. The system uses a computer-controlled robotics system to make it easier to generate pre-designed three-dimensional structures. By using this technology to produce the tubular structure, it was possible to examine its biological properties as explored by itoh M [221]. They created 500 MCSs (2.5 × 104 cells per MCS) using a “Bio-3D printer” which were composed of scaffold-free tubular tissues with an inner diameter of 1.5 mm formed of human umbilical vein endothelial cells (40%), human aortic smooth muscle cells (10%), and typical human dermal fibroblasts (50%). The tubular tissues were cultivated in a perfusion system before being implanted into the abdominal aortas of F344 nude rats. Following implantation, for five days, endothelial cells were seen.

Additionally, granular, microgel-based materials have gained attention as potential tissue-engineering scaffolds due to their inherent porosity, which can encourage cell penetration. It can be challenging to adapt these materials for 3D bioprinting while preserving enough void space to facilitate cell migration, as the rheological characteristics that define printability are primarily affected by microgel packing and void percentage [222]. Seymour AJ proposed a decoupling printability and void fraction method by combining UV-cross-linkable gelatin methacryloyl (GelMA) microgels with sacrificial gelatin microgels to generate composite inks [223]. Inks with an apparent viscosity of more than 100 Pas have been shown to have the rheological qualities necessary to print multilayered structures employing extrusion in air. This corresponds to microgel concentrations of less than 5 weight percent. The void fraction can be tuned from 0.20 to 0.57 by altering the ratio of GelMA to sacrificial gelatin microgels while keeping the overall concentration of the inks constant at 6 wt%. Human umbilical vein endothelial cells (HUVECs) implanted into printed constructs have also been reported to migrate through granular inks in a void fraction-dependent manner. Therefore, the microgel ink family has great potential for cell infiltration-based applications, including tissue engineering and 3D printing.

Hence, the design space for creating geometrically complex tissue scaffolds utilizing hydrogels with mechanical qualities similar to genuine tissues and organs in the human body has enlarged due to recent advancements in integrated bioprinting. Soft biomaterials can be embedded in a thermoreversible support bath at sizes ranging from millimeters to centimeters using techniques such as freeform reversible embedding of suspended hydrogels (FRESH) printing. By FRESH bioprinting a full-size model of an adult human heart from patient-derived magnetic resonance imaging (MRI) data sets, Mirdamadi E and his colleagues were able to widen the range of printable sizes [224]. To simulate the elastic modulus of cardiac tissue, they used alginate as the printing biomaterial. FRESH-printed alginate has been demonstrated to produce mechanically adjustable and suturable models and deliver high print fidelity on a budget-friendly printer platform. This illustrates the feasibility of the large-scale 3D bioprinting of soft hydrogels with FRESH and the production of heart tissue structures with potential future applications in surgical planning and training.

Furthermore, horseradish peroxidase (HRP)-mediated extrusion bioprinting has a lot of potential for applications in tissue engineering and regenerative medicine. However, they typically encounter problems with print quality and durability when using low-viscosity inks. Kotani T addressed this issue by coming up with a method to extrude support material and bioinks alternatively. Both the bioinks (cells, HRP, and phenolated polymers) and the support material contained hydrogen peroxide (H_2_O_2_) [225]. The support material, which also prevented the collapse of the constructs, provided the H_2_O_2_ needed to aid in enzymatic activity. A support material containing 10 mM of H_2_O_2_ was used to print 3D objects with height and intricacy using low-viscosity ink containing 10 U/mL of HRP and 1.0% *w*/*v* of phenolated hyaluronic acid (HA-Ph). Murine fibroblasts (10T_1/2_) showed a survival rate of over 90% after the printing procedure, and their morphology and rate of proliferation were comparable to those of untreated cells. After 14 days, human hepatoblastoma (HepG_2_) cells in the printed structures developed larger spheroids. On structures printed with phenolated gelatin and HA-Ph inks, the 10T_1/2_ cells could adhere and grow. These results demonstrate the great potential of HRP-mediated extrusion bioprinting for tissue engineering.

## 4. Applications of 3D Printing in the Medical Domain

The amalgamation of nanoparticles with 3D-printed polymers has arisen as a beneficial technique for diverse applications in the realm of medicine. Nanoparticles possess distinctive characteristics, including a notable ratio of surface area to volume, elevated reactivity, and the capacity for adjustment, rendering them well suited for the advancement of sophisticated medical apparatuses. The amalgamation of nanoparticles and 3D-printed polymers has promise in yielding functional materials with unique properties that may be tailored to specific biological applications. The following instances exemplify the practical implementations of polymer nanocomposites fabricated via 3DP.

### 4.1. Mechanical Applications

Mechanically improved implants are a key use case for 3D-printed polymers containing nanoparticles in medicine. Polymers with graphene, carbon nanotubes, or HA nanoparticles have better mechanical characteristics. These materials are utilized in the manufacturing of bone screws, prosthetic joints, and dental implants. By using 3D printing technology, nanoparticle dispersion may be precisely controlled, leading to enhanced mechanical qualities and a decreased chance of implant failure. The mechanical uses of polymers and their composites in medicine include the following.

i *Orthopedic implants:* Orthopedic implants have been 3D-printed employing polyethylene, polycarbonate, or polyetherketone (PEEK). These materials have high yield strength, durability, and wear and fatigue resistance. Incorporating carbon fibers or ceramics in polymers can improve their durability and render them suitable for applications requiring load support.
ii *Cardiovascular devices:* Cardiovascular devices, such as stents, pacemakers, and heart valves, often include polymers or their composites 3DP processes. These devices need materials that possess exceptional mechanical qualities, such as elevated strength, flexibility, and durability. Composites integrating silicon polyurethane and PTFE, along with metallic materials or ceramics, can enhance their durability [226].
iii *Tissue engineering:* Polymer composites act as scaffolds to support cell development and tissue regeneration. It is vital to investigate the mechanical characteristics of these materials to guarantee tissue’s appropriate functioning and its successful integration with surrounding tissues. Polymers such as polylactic acid (PLA), polyglycolic acid (PGA), or their copolymers (PLGA) have usually been employed in tissue engineering [226]. The incorporation of HA or silk fibers could potentially enhance the strength and flexibility of these polymeric materials [227].
iv *Drug delivery devices:* Implants, microparticles, and nanoparticles are all examples of polymer- or polymer-composite-based drug delivery methods [228]. Materials with specific mechanical qualities, such as drug-controlled release, biodegradability, and mechanical stability, are needed for these devices. Polymers such as PLA, PGA, or copolymers (PLGA) are often used in drug delivery [229].
v *Prosthetics:* Utilizing polymers and their composites, prosthetics are manufactured, including artificial hands, feet, and limbs. The devices mentioned earlier require materials with exceptional mechanical properties, such as strength, flexibility, and durability. PEEK, PMMA, and polyethylene (PE) are just a few of the polymers that have been widely used in 3DP.
vi *Surgical instruments:* Polymers such as nylon, polypropylene, and PEEK have widespread usage across various applications. Mechanically robust components must be employed to 3DP for optimal performance. These properties should include high strength, stiffness, and longevity.
vii *Diagnostic imaging:* In diagnostic imaging devices such as X-ray films, MRI coils, and ultrasound transducers, polymers composites are also employed. The aforementioned devices necessitate materials possessing distinct mechanical characteristics, including but not limited to electrical conductivity, flexibility, and acoustic impedance. Polymers, i.e., polyvinyl chloride (PVC), polystyrene, and PEEK, are frequently utilized [230].
viii *Wound care:* Dressings, bandages, and sutures use polymers and their composites. The aforementioned materials necessitate distinct mechanical characteristics such as pliability, permeability, and assimilation capacity. The incorporation of PTFE, polyurethane, or chitin with hydrogels or antimicrobial agents may enhance the strength and rigidity of these composites.
ix *Rehabilitation:* Rehabilitation applications commonly employ polymers and their composites, including but not limited to splints, braces, and supports. The successful deployment of these devices necessitates the utilization of materials that possess specific mechanical characteristics, including adaptability, toughness, and resilience. The utilization of polymers, namely nylon, PEEK, and polyethylene, is prevalent in various applications [231].

To summarize, orthopedic implants, cardiovascular devices, tissue engineering, dental applications, and drug delivery devices are all mechanical uses of polymers and their composites in the medical area. Continuous study and development in this subject will probably lead to even more spectacular breakthroughs in the future.

### 4.2. Drug Delivery Applications

Drug delivery is an additional area within the medical profession that shows promise for the use of 3D-printed polymers integrated with nanoparticles [232]. Nanoparticles, such as Au, Ag, and Fe_2_O_3_, have been integrated into polymer matrices in order to fabricate materials that are well suited to precision drug delivery purposes [233]. These materials can release pharmaceuticals based on pH, temperature, and magnetic fields. Improved medication release profiles and more accurate drug administration are possible because of 3D printing’s ability to precisely manage nanoparticle dispersion. Some benefits of 3D-printed polymers containing nanoparticles for use in medical-related medication delivery applications include the following.

i *Precise drug delivery:* The capacity to precisely regulate medication release is a major benefit of 3D-printed polymers containing nanoparticles for use in drug delivery. Experts may modify the structure, shape, and chemical composition of drug-containing nanoparticles to regulate their release. This may increase a treatment’s efficiency while decreasing the potential for adverse effects.
ii *Personalized medicine:* Drug delivery systems tailored to individual patients can be easily done via 3DP. Researchers have developed 3D models according to patients’ anatomy using imaging data, allowing them to better design medication delivery systems that are suited to their particular requirements [234]. This may enhance treatment results and lower the possibility of negative side effects.
iii *Reduced dosage:* Drugs may be released gradually over time with the use of 3DP drug delivery devices, which eliminates the need for several doses and lowers the total dosage needed. This may increase patient compliance and lessen the possibility of negative effects from excessive dosages.
iv *Cost-effective:* Three-dimensional printing in the pharmaceutical industry might drastically cut down on medication delivery system costs. Eliminating processes such as injection molding and machining might decrease manufacturing costs and boost healthcare accessibility.
v *Versatile:* The use of 3DP enables the fabrication of drug delivery systems encompassing a diverse array of forms and sizes. The adaptability of this characteristic might prove to be very advantageous in scenarios where targeted medication delivery to precise anatomical sites is required.
vi *Controlled release:* Drugs may be released through 3DP drug delivery devices in response to biological stimuli such as changes in pH or temperature [235]. This may increase a treatment’s efficiency while decreasing the potential for adverse effects.
vii *Targeted drug delivery:* The use of nanoparticles in conjunction with 3DP polymers enables the precise targeting of certain cells or tissues inside the human body. Hydrogel scaffold 3D-printed with incorporated nanoparticles of iron oxide, when exposed to a magnetic field, can attract and retain stem cells, which can be used to repair damaged tissues [235].
viii *Improved bioavailability:* Nanoparticles containing polymer may increase pharmacological bioavailability, allowing lower doses and fewer side effects. A microneedle patch composed of polymeric material can be fabricated via 3DP and that contains nanoparticles of the anti-cancer medication doxorubicin. The transdermal patch may be administered topically, facilitating the localized administration of medication and enhancing its absorption into the bloodstream. This method improves the delivery of medication and bioavailability [236].
ix *Extended release:* Incorporating nanoparticles into 3DP polymers allows for controlled medication release over a prolonged period of time. A stent was infused with the medication paclitaxel, combining polycaprolactone (PCL) and poly(lactic-co-glycolic acid) (PLGA), via 3DP [237]. Restenosis may be prevented using a medication that is slowly released from the stent over a period of weeks.
x *Customization:* The customization of medication delivery systems to satisfy the specific requirements of individual patients is made possible via 3DP. An insulin device made via 3DP for patients with type 1 diabetes may be molded to the patient’s anatomy and made from a biocompatible polymer containing insulin nanoparticles [238].

Popular materials often used in drug delivery systems developed via 3DP include PCL, PLGA, PEG, and hydrogels, as well as nanoparticles such as iron (III) oxide, gold, and silver [239]. These materials may be used in conjunction with 3DP to develop pharmaceutical delivery systems that exhibit improved efficiency, reduced bad effects, and more customization. The advantages included in this context encompass focused medication administration, personalized therapy, reduced dosages, decreased expenses, enhanced adaptability, and improved control over the drug’s release. The utilization of 3D printing’s distinctive attributes by researchers and producers may contribute to the improvement of patient outcomes and the advancement of medical science via the development of innovative drug systems.

### 4.3. Regeneration Applications

Three-dimensionally printed polymers with nanoparticles have also shown great promise in the field of tissue engineering. Nanoparticles such as carbon nanotubes and graphene have been integrated with polymers to develop materials that mimic natural tissues’ mechanical and electrical properties. These materials are used to create scaffolds that can support the growth and differentiation of various cell types. Three-dimensional printing technology allows for the precise control of nanoparticle distribution within the scaffold, resulting in improved tissue regeneration and repair. Hence, tissue engineering involves creating functional biological tissues by combining cells, biomaterials, and biochemical cues [240]. Three-dimensional printing has enabled the fabrication of complex and customized scaffolds with high accuracy and precision, making it a promising tool for tissue engineering applications [241]. The use of 3D-printed polymers with nanoparticles in tissue engineering offers several advantages, including the following:

i *Enhanced scaffold properties:* Incorporating nanoparticles into the polymer matrix can improve a scaffold’s mechanical strength, stiffness, and degradation properties. For example, the addition of silica nanoparticles to a polycaprolactone (PCL) scaffold improved its mechanical properties, making it suitable for bone tissue engineering [242]. Dante Ronca and his colleagues used AM to create biodegradable and nanocomposite scaffolds for bone tissue regeneration [243]. These scaffolds were made of poly(-caprolactone) (PCL) reinforced with hydroxyapatite nanoparticles. The architecture and the addition of HA influenced the mechanical performances of the built scaffolds. The construction process affected the scaffolds’ mechanical attributes. It was discovered that scaffolds with a 0/90° pattern had a higher compressive modulus and higher maximum stress than those with 0/60/120° and 0/45/90/135° patterns because a greater contact area (i.e., fused area) correlated to a smaller amplitude of the deposition angle (from 0/90° to 0/45/90/135°). Fibers placed using a lay-down pattern of 0/45/90/135° showed reduced stiffness [243].
ii *Controlled release of growth factors:* Growth factors are important for promoting cell proliferation and differentiation in tissue regeneration. Incorporating nanoparticles into a polymer matrix can help control the release of growth factors over a prolonged period. For example, a study used gelatin-based scaffolds loaded with chitosan nanoparticles to deliver vascular endothelial growth factor (VEGF) in order to promote angiogenesis in wound healing [244]. Similarly, 3D-printed polylactic acid (PLA) scaffolds with the addition of polyethyleneimine-coated iron oxide nanoparticles were used to encapsulate and control the release of bone morphogenetic protein-2 (BMP-2), a growth factor that promotes bone regeneration [245].
iii *Biomimicry:* Three-dimensionally printed polymers with nanoparticles can be designed to mimic the structure and properties of natural tissues. For example, a study used a blend of PCL and hydroxyapatite nanoparticles to fabricate a scaffold for cartilage tissue engineering. The resulting scaffold exhibited similar mechanical and biological properties to native cartilage tissue [246].
iv *Customization:* Three-dimensional printing allows customized scaffolds to be fabricated with precise control over their shape, porosity, and interconnectivity. This enables the creation of patient-specific implants that can integrate seamlessly with the surrounding tissue. For example, a study used 3D-printed PCL scaffolds loaded with gelatin nanoparticles for spinal cord injury repair [247].
v *Reduced cost and time:* Three-dimensional printing enables the rapid prototyping of scaffolds, reducing the time and cost associated with traditional manufacturing methods. This makes it a cost-effective solution for tissue engineering applications.
vi *Improved cell adhesion and proliferation:* The addition of nanoparticles can also improve a scaffold’s ability to support cell adhesion and proliferation, which are important steps in tissue regeneration. For example, researchers have developed 3D-printed PCL scaffolds with the addition of graphene oxide nanoparticles, which improved the scaffolds’ ability to support the adhesion and proliferation of human mesenchymal stem cells, which are important cells for bone tissue regeneration [248].
vii *Personalized Tissue Engineering:* Three-dimensional printing allows for the creation of patient-specific tissue engineering scaffolds, which can be tailored to an individual’s unique anatomy and pathology. Three-dimensional printing has been used to create personalized nasal implants for patients with nasal defects made from a polymer/nanoparticle composite material [249].
viii *Enhanced angiogenesis:* The formation of new blood vessels is critical for the growth and survival of engineered tissues. The addition of nanoparticles to the polymer scaffold can promote angiogenesis by enhancing the release of angiogenic growth factors or by creating a microenvironment that supports the growth and migration of endothelial cells. For example, researchers have developed 3D-printed PCL scaffolds with the addition of gold nanoparticles, which improved the angiogenic properties of the scaffold and enhanced the formation of blood vessels [250].
ix *Improved antibacterial properties:* Adding nanoparticles with antibacterial properties, such as Ag nanoparticles, can help prevent bacterial infections in tissue engineering applications [251]. Three-dimensionally printed PCL scaffolds with the addition of Ag nanoparticles have shown excellent antibacterial properties against Staphylococcus aureus, a common bacteria associated with infections in tissue engineering [252].
x *Drug screening:* Three-dimensionally printed polymer with nanoparticles can also be used as a platform for drug screening, allowing for the testing of drugs on engineered tissues in a controlled environment. Three-dimensionally printed PCL scaffolds with the addition of magnetic iron oxide nanoparticles were used to create a magnetic field that could be used to control the direction of cells and drug particles within a scaffold [253].
xi *Nerve regeneration:* Three-dimensionally printed polymers with nanoparticles can also be used for nerve regeneration. Three-dimensionally printed scaffolds with the addition of graphene oxide nanoparticles showed improved electrical conductivity and promoted the differentiation of neural stem cells into neurons [254].

These are just a few examples of the many applications of 3DP polymers with nanoparticles in tissue engineering. The ability to precisely control the composition and properties of a scaffold through the addition of nanoparticles holds great promise for the development of more effective tissue engineering therapies. Examples of the materials used in 3D-printed polymers with nanoparticles for tissue engineering applications include PCL, polylactic acid (PLA), gelatin, chitosan, hydroxyapatite, and silica nanoparticles [255].

### 4.4. Diagnostic Applications

The combination of nanoparticles and 3DP polymers has demonstrated significant potential in the field of diagnostics. Polymers have been combined with nanoparticles such as Au, Ag, and magnetic particles, i.e., Ni, Zn, and Mg, to create advanced materials suitable for imaging and biosensing purposes [256]. These materials can be designed to detect specific molecules or cells, making them ideal for various diagnostic applications. Increased sensitivity and specificity may be achieved via 3DP technology since it allows for the exact monitoring of the dispersion of particles inside the material. The use of 3D-printed polymers with nanoparticles in diagnostic applications in the medical field has several advantages and benefits, some of which include the following:

i *Personalized diagnostics*: Three-dimensional printing technology enables the production of patient-specific diagnostic devices to detect biomarkers or analyze samples in real time. This personalized approach can enhance the accuracy of diagnostics and enable more targeted treatments. Three-dimensionally printed microfluidic systems containing iron oxide nanoparticles may identify heart attack biomarkers promptly [257].
ii *Rapid diagnostics:* Devices for quick sample analysis and real-time findings may be developed using 3DP polymer with nanoparticles. Microfluidic devices, including carbon nanotubes, have been successfully 3D-printed, allowing for quick bacterial detection [258].
iii *Cost-effective diagnostics:* Three-dimensional printing technology now allows for the rapid and low-cost production of diagnostic instruments. Low-cost, rapid-prototyping 3D-printed diagnostic strips include silver nanoparticles for detecting microbial and viral illnesses.
iv *Improved accuracy:* The ability to create 3D-printed diagnostic devices with complex geometries and precise dimensions can improve the accuracy of diagnostic testing. A 3DP microfluidic approach is based on Fe_2_O_3_ nanoparticles for very accurate and sensitive detection of RBCs infected with malaria [259].
v *Customizable design:* Three-dimensionally printed diagnostic devices allow for the fabrication of devices that may be adapted to individual diagnostic requirements via the customization of design and material qualities. In order to detect various analytes, such as glucose, urea, and cholesterol, scientists have created a 3D-printed biosensor that incorporates graphene oxide nanoparticles [260].
vi *Sensitive and specific detection:* The addition of nanoparticles to the polymer scaffold can improve the sensitivity and specificity of diagnostic devices. The incorporation of Au nanoparticles to a 3D-printed biosensor allows it to detect low quantities of a cancer biomarker with great sensitivity and specificity.
vii *Real-time monitoring:* A 3D-printed microfluidic device containing magnetic nanoparticles helps monitor the growth of microbes and their response to drugs in real time.

Improved accuracy, adaptability, an adjustable design, accurate and focused detection, and real-time monitoring are just a few of the benefits gained by using 3D-printed polymers with nanoparticles in medical diagnostics. Three-dimensionally printed polymer composites with nanoparticles embedded in them might revolutionize medical diagnosis [261]. This significant advancement holds the promise of facilitating the development of personalized, minimally intrusive, highly responsive, expeditious, and cost-effective diagnostic instruments.

### 4.5. Wound Healing Applications

Within the field of wound healing, there have been notable advancements in the utilization of 3D-printed polymers that include nanoparticles, showcasing encouraging outcomes. Antibacterial polymers have been developed by adding nanoparticles, particularly Ag and ZnO. Wound dressings made from these materials help speed healing and protect against infection. The use of 3DP technology has led to increased wound healing properties (201) due to the ability to precisely manage the distribution of nanoparticles inside the material. In wound healing, 3D-printed polymers containing nanoparticles have several benefits. Some examples include the following:

i *Controlled drug release:* Three-dimensionally printed polymers with nanoparticles can be used to create wound dressings that release drugs or growth factors in a controlled manner. Chitosan nanoparticles can accelerate the healing of wounds when incorporated into 3DP polycaprolactone (PCL)/gelatin scaffolds [262]. This incorporation offers the advantages of increased antibacterial activity and the controlled release of growth factors.
ii *Customized wound dressings:* Three-dimensional printing enables the production of personalized wound dressings that accurately correspond to the size and shape of specific wounds. This phenomenon has the potential to result in enhanced wound covering and expedited healing durations.
iii *Reduced risk of infection:* Nanoparticles boost a polymer scaffold’s antibacterial properties, reducing infection risk. Three-dimensionally printed PCL/gelatin scaffolds exhibited remarkable antibacterial efficacy against a variety of bacterial species when combined with Ag nanoparticles.
iv *Enhanced tissue regeneration:* Tissue regeneration is improved when scaffolds made from 3DP polymer containing nanoparticles are utilized to model native tissue architecture, i.e., HA nanoparticle-enhanced 3D-printed PCL/gelatin scaffolds for bone regeneration.
v *Customized wound dressings:* Customized wound dressings may now be printed in any size or form using 3DP technological advances. This may boost the dressing’s effectiveness and protect against infection.
vi *Enhanced drug delivery:* Improving medication distribution to the wound site by incorporating nanoparticles into the polymer scaffold may accelerate the healing process. Ag nanoparticles, incorporated into 3D-printed wound dressings, are antimicrobial and aid in wound healing.
vii *Reduced healing time:* By providing a framework that cells can grow and renew on, 3D-printed polymers containing nanoparticles might expedite the healing process. The improved adherence of cells and proliferation, in turn, accelerate up the wound healing mechanism when Au nanoparticles are integrated into 3D-printed polycaprolactone (PCL) scaffolds.
viii *Reduced scarring:* 3D-printed polymers with nanoparticles can also reduce scarring by promoting healthy tissue formation. New blood vessel growth was stimulated and inflammation was reduced via 3DP PCL scaffolds loaded with hyaluronic acid nanoparticles.

By delivering individualized, improved, and targeted therapy for wound healing, 3D-printed polymers with nanoparticles have the potential to increase positive clinical results. Thus, 3D-printed polymers with nanoparticles in wound healing applications may provide personalized wound dressings, controlled medicine release, reduced infection risk, and enhanced tissue regeneration.

### 4.6. Biocompatibility Enhancement

The biocompatibility of 3DP polymers may be enhanced via the incorporation of particles. Nanoparticles such as titanium dioxide and hydroxyapatite have been integrated with polymers to develop materials that promote cell growth and reduce inflammation [263]. The usage of these materials in the creation of implants and scaffolds demonstrates their compatibility with the human body. By controlling the distribution of nanoparticles inside a material in fine detail using 3D printing technology, biocompatibility may be improved.

i *Reduced inflammation and toxicity:* Nanoparticles that minimize inflammation and toxicity, such as silver or gold, may be used to increase the biocompatibility of polymers. In vitro studies have demonstrated that 3D-printed scaffolds incorporating Au nanoparticles reduce inflammation and increase cell proliferation. Three-dimensionally printed polymer scaffolds containing GO display lower cytotoxicity than that of traditional medical device materials.
ii *Improved mechanical properties:* Polymer materials may find more use in the medical field if nanoparticles are included to enhance their mechanical qualities. Three-dimensionally printed poly(lactic-co-glycolic acid) (PLGA) scaffolds’ endurance and compatibility were enhanced via the inclusion of MgO particles.
iii *Enhanced drug delivery:* Targeted medicine distribution to specific bodily areas may also be accomplished using 3D-printed nanoparticle polymers. Mesoporous SiO_2_ nanoparticles may be integrated into 3DP hydrogels to provide a prolonged release of medicines and other therapeutic drugs.
iv *Tissue regeneration:* Three-dimensionally printed polymers with nanoparticles can also be used for tissue regeneration applications. In vivo, 3D-printed chitosan scaffolds containing Ag nanoparticles boosted antibacterial characteristics and tissue regeneration.
v *Biodegradability:* Iron oxide and calcium phosphate nanoparticles may improve polymer biodegradability, making them acceptable for implanted medical devices. The inclusion of Fe_2_O_3_ nanoparticles into 3D-printed polycaprolactone (PCL) scaffolding enhanced the rate of biological degradation in culture.
vi *Improved implant integration:* Three-dimensionally printed polymers with nanoparticles can enhance the integration of implants with adjacent tissue. For instance, the addition of bioactive glass nanoparticles improved an implant’s compatibility and facilitated its integration with adjacent tissue.

This advancement offers a range of potential advantages, such as mitigating inflammation and toxicity, enhancing mechanical qualities, facilitating drug transport, promoting tissue regeneration, and enabling biodegradability.

### 4.7. Surgical Training Applications

Three-dimensionally printed polymers with nanoparticles can also be used for surgical training. Materials for 3D-printed anatomical models have been developed by combining nanoparticles such as barium sulfate and iodine with polymers. Surgeons may use these models to design and perform complex procedures on fake patients before attempting them on real people. The precise control of nanoparticle distribution throughout the material made possible by 3DP technology allows for anatomically accurate replicas to be made. The use of 3D-printed polymers with nanoparticles in surgical training applications has several advantages and benefits, including the following:

i *Realistic models:* A 3D-printed polymer with nanoparticles can be used to create highly realistic surgical models that closely mimic the physical properties of human tissue. This facilitates enhanced surgical training and preparation, resulting in improved patient outcomes.
ii *Customization:* The utilization of 3DP permits the fabrication of customized surgical models and simulators, thereby facilitating the customization of these instruments to meet the specific needs of individual surgeons and medical trainees. The efficacy and efficiency of surgical training courses may be enhanced via the adoption of this technique.
iii *Cost-effective:* Traditional surgical training methods can be expensive and time-consuming. Three-dimensional printing eliminates the need for costly surgical models and cadavers.
iv *Risk reduction:* Three-dimensionally printed surgical models and simulators decrease surgical complications and mishaps. They improve patient safety and reduce the cost of healthcare.
v *Improved surgical planning:* Surgeons may design and perform difficult surgeries using 3D-printed surgical models. This can lead to more precise and efficient surgeries.

Laparoscopic surgery, orthopedic surgery, and neurosurgery training models are a few real-world examples of 3D-printed surgical teaching models. Different complexity and detail levels may be produced for these models, providing for a variety of training settings and ability levels. Adding nanoparticles to the polymer matrix can also enhance the mechanical properties and durability of the models, increasing their usefulness for surgical training.

### 4.8. Neural Tissue Engineering

The combination of 3D printing with nanoparticles has shown promise in the area of brain tissue engineering. Carbon nanotubes and graphene, for example, have been combined with polymers to generate materials that can imitate the electrical characteristics of brain tissue. The materials indicated above are used in the creation of scaffolds, which are critical in providing structural support for the development and differentiation of brain cells. Three-dimensional printing technology enables the precise modification of nanoparticle dispersion inside a scaffold, resulting in better brain regeneration and repair. The use of a 3D-printed polymer with nanoparticles in neural tissue engineering has several advantages and benefits, including the following:

- *Precision in creating neural tissue structures*: Researchers can now develop intricate, patient-specific models of neurological disorders and injuries using 3DP, which allows for the accurate manufacturing of neural tissue structures.
- *Enhanced neural tissue growth:* It has been shown that incorporating nanoparticles into the polymer framework enhances brain tissue development and regeneration. Adding GO nanoparticles to 3D-printed PCL scaffolds promotes neural stem cell proliferation and axon outgrowth.
- *Customizable scaffolds:* The application of 3DP enables the customization of scaffolds that closely mimic the intricate structures of brain tissue. This may enhance the scaffold’s compatibility and integration with the surrounding brain tissue, aiding in tissue repair and regeneration.
- *Brain implants*: The creation of brain implants that may link to the brain and provide therapeutic benefits has shown promise when 3D printing technology and polymer materials containing nanoparticles are combined. Three-dimensional printing may be used to create a neural implant for focused brain stimulation to treat epilepsy.
- *Drug delivery*: Targeted medication administration to the brain is another potential use of 3D-printed polymers with nanoparticles. Drug-releasing microdevices created on a 3D printer may be implanted in the brain and activated via external stimuli such as light or heat. The integration of drug-loaded nanoparticles into a 3D-printed scaffold may effectively improve treatment effectiveness and reduce the occurrence of undesirable effects.

**Figure 11:**
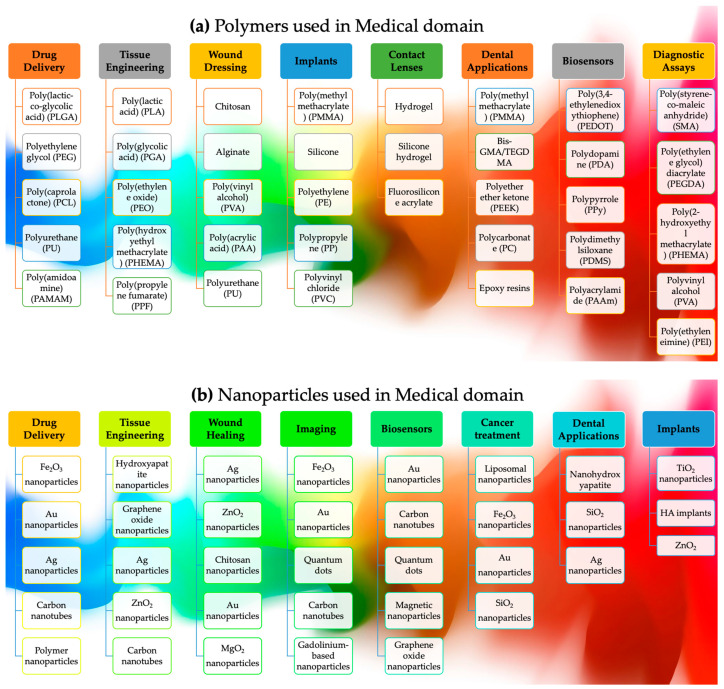
Summary of the essential applications of (**a**) polymers and (**b**) nanoparticles in various medical dimensions using 3D printing.

In general, the utilization of 3D-printed polymers integrated with nanoparticles in the field of neural tissue engineering has promise in enhancing the comprehension of neurological ailments and injuries, as well as facilitating the creation of more efficacious therapeutic interventions. The amalgamation of nanoparticles into polymers has resulted in the creation of functional materials that exhibit distinctive characteristics and may be customized to suit certain biological purposes. The use of 3DP technology enables the exact manipulation of nanoparticle dispersion inside a substance, leading to enhanced mechanical, medication administration, tissue engineering, and diagnostic characteristics. The potential applications of 3DP polymers included with nanoparticles in the medical domain are vast and boundless, owing to the continuous progress and exploration in research and development. The continuous investigation and advancement in this domain are expected to provide more remarkable advancements in the forthcoming years. Table 1 and Figure 11 summarize the benefits of 3D-printed polymers and their combination in a variety of contexts. The limited number of polymer alternatives available is a significant issue in 3D printing materials. Extrusion and injection molding methods can handle hundreds of polymer constituents [264].

### 4.9. 3D-Printed Biosensors

Three-dimensional printing enables the decentralized production of on-demand, low-cost sensors and actuators for prototyping in point-of-use biosensing systems. Although this field of study is still in its early stages, the 3D printing of bioanalytical platforms has immense promise in various domains, including electrochemical and optical devices [285]. The early discovery of the luteotropic hormone prolactin (PRL) has a lot of treatment value in chemistry and biology. It can help avoid dangerous diseases such as prolactinoma, hyperprolactinemia, hypothyroidism, menstrual instability, galactorrhea, and infertility. Conventional ways of finding PRL include enzyme-linked immunosorbent tests, polymerase chain reactions, radioimmunoassays, and liquid-chromatography-linked tandem mass spectrometry. However, these methods are hard to use because they are expensive, take a long time to measure, and use toxic material [286]. To solve these problems, 3D-printed metal nanoparticles and polymeric plates comprising carbon nanotubes, graphene, polymer films, gold/ or platinum/palladium nanoparticles, magnetic beads/particles, and metal-organic frameworks are used for accurate and stable PRL detection [287].

Besides this, the COVID-19 pandemic has focused a lot of emphasis on the need for viral detection. Gustavo Martins demonstrated the creation of an immunosensor consisting of carbon black and polylactic acid (PLA) to identify Hantavirus Araucaria nucleoprotein (Np) caused by coronavirus (SARS-CoV-2) [288]. The recognition biomolecule was connected directly to the surface of the filament using N-(3-Dimethylaminopropyl)-N′-ethylcarbodiimide hydrochloride and N-Hydroxysuccinimide (EDC/NHS). The findings revealed that the device could measure the quantity of Hantavirus Araucaria nucleoprotein (Np) in the range of 30 μg mL^−1^ to 240 μg mL^−1^ with a detection limit of 22 μg mL^−1^. In addition, the suggested immunosensor effectively detected viruses in human blood samples.

Similarly, 3DP technology could be used to make (bio)sensors that can be used to test different body fluids. Cardoso RM used FDM to build (bio)sensing platforms from widely available polylactic acid fibers mixed with graphene (G-PLA) [289]. Chronoamperometry was used to make the biochemical glucose detector and put it on the G-PLA surface to identify glucose in blood plasma. The process of making an enzyme immobile by binding it with glutaraldehyde works better if the polymeric binder has oxidized groups [290]. New materials, such as polylactic acid (PLA) and acrylonitrile butadiene styrene (ABS), showed how flexible and valuable FDM3D printing is for making electrochemical sensors using commercial graphene/PLA or carbon black/PLA filaments [291]. It has been shown that 3D-printed surfaces can be changed chemically to make better electrochemical sensors. For example, the electrodeposition of Prussian blue nanoparticles was used to analyze milk and cosmetic samples without interfering with species common in milk, cosmetic, and biological samples [292]. It has been shown that enzymes can be added to 3D-printed surfaces to make electrochemical biosensors. However, this new addition only showed how biosensing detection could be done, not how it could be used to analyze real-world, complex samples.

Hence, 3DP technology opens up exciting opportunities in many areas [293]. In electrochemistry, 3D printing has been used to make personalized 3D-printed electrodes that can be used as a base to build devices for biosensing, making energy, and storing it, i.e., magnetoelectric wearable sensors [294] and a FeCuNbSiB/PVDF sensor. Similarly, 3DP graphene/polylactic acid (PLA) electrodes bind horseradish peroxidase (HRP) to make an enzyme-based detector that can detect hydrogen peroxide [295]. Gold nanoparticles are part of a system to prove and help with the diffuse movement of electrons. Using 3D printing technology, this work shows a new way to make third-generation electrochemical biosensors, which could be used in the environmental and medical fields [296].

## 5. Drawbacks

The materials’ mechanical strength might be challenging for polymers exhibiting structural flexibility. It is feasible to obtain different degrees of reinforcing effectiveness in polymers by including various plastic particles in the mix [297]. Significant variations exist between particles containing organic, inorganic, and metallic elements regarding their stiffness and strength [298]. Nanocarbons typically have strengths and intrinsic moduli ranging between 50 and 200 GPa, depending on their size and composition. Additional factors that influence the transmission of particle characteristics to the polymer matrix include particles’ size and surface area. The capacity of these particles to interact with nearby polymer chains rises as the size of the particles decreases. The effectiveness of composites is affected by particle sizes and configurations; according to composite mechanics, stress is transmitted to molecules in the composite material [299]. Nanotubes that are longer have a stronger capacity to transfer strength when compared to nanotubes that are shorter in length [300].

The massive market for 3D printing, the massive number of polymers used by the technology, and the rapid advancement of polymeric materials have created a significant issue in polymer recycling and the circular economy [301]. This issue has implications for both the circular economy and polymer recycling. Aside from that, the rapid development of 3D printing technology has resulted in an enormous accumulation of polymeric waste from individual and industrial printing operations, notably in the case of thermoset materials [302]. Polymers, hybrids, and composites that are improperly disposed of in landfills or incinerators pollute the environment, lakes, rivers, and the earth. An effective strategy for increasing reliability, recyclability, and repairability using novel recyclable materials, as stated in Table 2, is to take advantage of the support and broad variety of 3D printing platforms available.

The recycling of polymers has already begun to have an influence on three-dimensional printing. PET, HDPE, PVC, LDPE, PP, and PS are just a few of the thermoplastics that may be recycled via FDM. Filament manufacture and FDM are the most prevalent recycling processes. Due to their mechanical reinforcing in polymer matrixes and high degradability, biofuel polymers such as lignocellulosic biomass are easy to recycle [303]. This is owing to their great degradability and biomechanical reinforcement in polymer matrixes, which make them extremely recyclable. The effectiveness and adaptability of thermoset recycling in wind turbines, automobiles, and other applications have been demonstrated and aerospace industries [304]. Due to their mechanical strength and chemical resistance, they are not appropriate for shredding, pelletizing, or sorting, making recycling them a challenge. They are very difficult to compound once they have been extracted and compounded. According to the EPA, the time required to identify and isolate individual plastics from waste mixtures is also the most costly component of plastic recycling. In the future, the reuse of these components in FDM and the likelihood of damage during heating may cause issues in recycling.

Another technology for printing sustainability that uses thermoset polymers is self-repairing materials. This technique is also used to print food packaging. The self-healing mechanism employs both extrinsic and intrinsic self-healing processes. It is used in conjunction with the extrinsic approach, which stores healing chemicals in micron canisters released when the canisters are destroyed. In the intrinsic approach, reversible covalent or noncovalent bonds are employed. These bonds are reconstructed in reaction to environmental stimuli, such as heat or cold (e.g., heat, electricity, light, moisture, and pH). Extrinsic healing structures benefit from 3D printing, a highly strong instrument in the healing process. The techniques of stereolithography, digital light processing, and inkjet printing, for example, may all be utilized to effectively generate vascular networks inside a polymer matrix, which can then be employed to encourage the passage of the healing agent. Flow rates and healing efficiency are affected by various factors, including optimizing the topology, establishing the gradient structure, and designing microfluidics. PMMA with urea–formaldehyde microcapsules, for example, has increased healing effectiveness by 87% despite the PMMA level being just 5% [305]. When it comes to 3D printing with recyclable polymers and composites, the options are almost limitless; nevertheless, the current recycling system sets constraints on material types, such as FDM filaments and DIW gels, among others.

There are many advantages to using 3D-printed polymers with nanoparticles in medical applications. There are also some potential disadvantages to consider. Here are some of the main drawbacks.

- *Material properties:* The size, distribution, and bond strength of nanoparticles in 3D-printed polymer composites may affect their mechanical properties. This can result in unpredictable mechanical behavior and reduced reliability compared to conventionally manufactured parts.
- *Regulatory challenges:* The absence of specific regulations for the utilization of 3D-printed polymers containing nanoparticles in medical applications poses regulatory challenges. It is sometimes difficult to acquire regulatory clearance for innovative devices, which might raise questions about their usefulness and safety.
- *Cost:* Particularly when producing high-grade medical-grade materials and specialist equipment, 3D printing may be rather costly. Due to this, it may be challenging to compare the cost of 3D-printed medical apparatus to that of conventional manufacturing methods.
- *Limited material options:* The range of materials available for 3DP is currently limited compared to traditional production methods despite the potential use of various polymers and nanoparticles. This may restrict the design possibilities and functionality of 3D-printed medical devices.
- *Surface finish*: In comparison to parts created using traditional means, it is common for 3D-printed components to have a somewhat coarse surface texture. This might provide challenges in terms of cleaning and disinfection, especially in medical contexts where maintaining sterility is crucial.
- *Biocompatibility:* A broad spectrum of polymers and nanoparticles were recognized as biologically compatible. The long-term biocompatibility of 3DP components is still uncertain.
- *Size limitations*: The existing capabilities of 3D printing technology impose limitations on the dimensions of printable objects. This is especially problematic in contexts where bigger components, such as implants or prostheses, are needed, such as in medicine.
- *Durability:* Although 3D-printed components exhibit notable strength and durability, apprehensions arise over their sustained performance when they are subjected to settings including repetitive loading or exposure to severe environmental factors. The complex microstructural composition of 3D-printed components makes them more prone to failure or degradation over time.
- *Intellectual property:* Three-dimensional printing technology makes it easier to replicate existing medical devices or parts, which can raise concerns about intellectual property and patent infringement. This can create legal challenges for manufacturers and limit the incentives for innovation in the field.

The utilization of 3D-printed polymers with nanoparticles in medical applications presents numerous advantages, but it also entails certain drawbacks. The utilization of 3D-printed polymers with nanoparticles holds significant potential for medical applications.

Researchers may create medical devices that are safe, effective, and inventive by thoroughly weighing the pros and cons of this technology. These possible downsides and restrictions should be considered with the numerous potential benefits of employing 3D-printed polymers containing nanoparticles in medical applications. These dangers may be reduced by paying close attention to material choices, process optimization, and regulatory compliance while 3D printing medical items. Shrinkage is a significant concern for polymers that are either crystalline or semicrystalline [306]. Laser contact has the potential to produce depolymerization of polymers with melting temperatures that are near the degradation temperature as well—for example, perfluorinated polymers, with a melting temperature of 300 °C and degradation temperature of 320 °C [300]. As a result of this, only a limited number of high-performance polymeric temperatures (i.e., HDPE, PVA, or PP) and only a small number of engineering polymers can be processed utilizing filament-based 3DP technology (PI and PEEK). Viscous inks are particularly well suited to biomedical applications since they may be utilized to treat delicate materials and cells. Dissolving these materials necessitates specialized solvents and a high degree of customization [307].

Three-dimensional printing based on ink writing primarily concerns tissue scaffolds. Using a gel to print in vivo biomedical or biological research may be inefficient since it does not meet the stringent standards for biocompatibility and regulated biodegradability, which are essential in such research, as organic solvents do. There is still more work needed to achieve the full potential of smart materials and structures shortly despite substantial advances in 4D printing over the last decade. A majority of today’s 4D printing methods, including SLA, inkjet, FDM, and LDM, depend on individual layer deposition. The printed structures are layered or textured as a consequence of individual layer deposition in each of these technologies. Some of these three-dimensional printing technologies use a combination of casting, molding, stretching techniques, and other techniques. As a result, an integrated manufacturing process that incorporates many materials and numerous 3D printing methods on the same printing platform would be more powerful than standard 4D printing techniques. The second reason is that shrinking at the micro- and nanoscale in MEMS or NEMS needs higher-resolution printing (e.g., nano-manufacturing), and 4D printing is less efficient at submicron sizes than at large dimensions. The present state of nanoscale 3D printing is limited to a few technologies (e.g., EHD and 2/MPP), and new printing methodologies must be developed to advance the field. Other challenges must be solved soon, such as a lack of material possibilities and poor mechanical properties.

## 6. Conclusions

The progress in 3D printing technology and its medical applications have yielded numerous advantages and opportunities. The technology shows great potential in creating personalized prosthetics, implants, and organs that are customized to fit the unique anatomical features of patients. The use of 3D printing in constructing complex structures has significantly impacted the approach of medical professionals towards patient care. The expense of both the 3D printing technology and its components continues to be a barrier to the technology’s widespread application, particularly among less substantial healthcare establishments. The lengthy production process can also be a barrier, specifically in critical circumstances where devices need to be manufactured in a hurry. This holds particularly true during emergency situations. However, we expect that these limitations will diminish in significance as research and development endeavors progress. Undoubtedly, the medical industry can derive significant advantages from 3D printing despite the inherent drawbacks of its costly and time-consuming manufacturing process. These limitations can be overcome through ongoing research and development, enhancing the tool’s value as a crucial asset for the future of healthcare.

Besides this, 3D printing can achieve great performance without the need for mechanical assembly while simultaneously reducing material waste and waste disposal. The compatibility of manufacturing materials with one another may inspire new manufacturing mechanisms and processes. Different printing techniques, e.g., vat polymerization, are utilized in a number of methods, including SLA, 2PP/MPP, DLP, and CLIP, just to mention a few examples. The resolution of their printing is determined by the energy sources of light utilized, which may range in size between just a few 100 nm and 10 μm, depending on the application in which they are used. However, when it comes to most of these printing techniques, curing monomers is necessary; yet, including particles impacts how quickly the curing process proceeds. Jetting methods, such as direct inkjet, EHD jet, and binder jetting, are procedures that depend on jetting to generate outcomes. When using these technologies, the flow dynamics, rheology, and design of the printing nozzles all define the printing principle. It is possible to introduce nanoparticles throughout printing patterns using direct inkjet. The packing density of the particles is regulated by how concentrated the ink is used to deposit them. When it comes to manipulating nanoparticles at high resolutions, the EHD jet has shown to be quite effective. However, because of the small diameter of the printing nozzle and the high frequency with which it becomes clogged, it is challenging to achieve faster printing speeds than those now available. To be effective, vat polymerization and jetting-based 3DP both need photosensitive monomers to have certain compositional, optical, and rheological qualities in order to function. This holds particularly true during emergency situations. However, we expect that these limitations will diminish in significance as research and development endeavors progress. The monomer composition used in the curing process affects the curing kinetics, including factors such as functional groups, crosslinking, and photoinitiators. Greater 3D printing resolutions are reached as a result of the ability to precisely modify specific elements of the 3DP process (e.g., 2PP and EHD). Optical scattering, nanoparticle loading, and particle size distribution are all affected by the composite resins’ particle quality. The primary factors of particle quality impact these properties. The particles used in vat polymerization have a wide range of active compound admixtures for tissue engineering. The particles used within jetting-based printing for biomedical applications contain a comparable range of bioactive fillers. (e.g., Ag nanorods, ceramics, or carbon wires). Greater printing resolutions may be achieved by improving the compatibility of nanomaterials with certain 3D printing techniques and by gaining a better understanding of the multiphase phenomena that are occurring.

Extrusion-based 3D printing (3DP) methods, i.e., FDM and liquid deposition modeling (LDM), have sparked a lot of attention in the scientific community due to their simple installation and printing processes. The processability and quality of specimens in FDM are greatly influenced by the mechanical, thermal, and rheological properties of thermoplastic filaments. Although LDM may not provide the same level of advantages as FDM in relation to operational temperature, it does exhibit compatibility with a broader spectrum of macromolecules compared to what can be achieved with FDM. The majority of particle additions to FDM and LDM result in increased viscosity in the final product. Polymer chains and particles are orientated preferentially during the deposition of filamentary materials due to the shear flow created during the deposition of filamentary materials. Polymeric particles are used in powder bed fusion-based methodologies such as SLS to augment both the procedural efficacy and the ultimate product’s quality. Nanoparticles’ polymeric materials melt or soften when subjected to heat and then resolidify at room temperature. The sintering frame refers to the temperature range in which polymers may solidify (melt, crystallize, etc.) without being deformed in any way. The rheological viscosity, surface tension, and power are quantitatively assessed. The properties of the powder utilized in the printing procedure impact both the dispersion of laser-powder interactions and the uniformity of the printing process. The use of small nanoparticles may lead to high-quality printing when printing at fast speeds. In addition to causing powder aggregates to form due to Van der Waals forces, much smaller particles (<20 microns) can cause airborne dust clouds to form, which may impact the printing resolution. As a result of the intimate interaction between production techniques and material features, we looked into various issues and potential applications for 3DP nanomaterials. We concentrated on the efficient production variables in printing polymer/nanostructured particle materials and nanocomposite structures, particularly in printing polymer/particle nanocomposite structures. The materials utilized, the particulate suspensions or densities, the interfacial interactions, and the particle size distributions are all considered and optimizations when constructing high-performance composites. It was necessary to employ a variety of unique 3D printing applications in rapidly growing industries. The integration of future 3D printing technologies or novel processing processes with known 3D printing technologies has the potential to facilitate the production of intricate architectural designs and functional characteristics across various sizes and material compositions. We hope that this article’s overview of 3D printing will aid in the direction of future research into innovative advanced production systems or design patterns for the further, faster utilization of polymer composites, as well as particulate (for example, elevated printing resolutions and manufacturing speeds), and succeeding structures that are not currently possible using conventional manufacturing methods.

## Figures and Tables

**Table 1:** A variety of aspects of 3D printing technology, polymers, and their composite, along with their respective advantages.

Method	Year	Polymer	Powder	Benefits	Ref
FDM	2022	Polylactic acid (PLA)	Graphene oxide (GO)	Improved mechanical, thermal, and electrical properties of nanocomposites	[264]
FDM	2021	Nylon, polycarbonate, and PEEK	Glass, carbon, and aramid fibers	Enhanced mechanical and thermal properties of composites, improved printability and surface quality, and cost-effectiveness	[265]
Fused filament fabrication (FFF)	2020	Polyetheretherketone (PEEK)	Carbon nanotubes (CNTs)	Improved mechanical, thermal, and electrical properties of composites, enhanced printability, and reduction in defects and porosity	[266]
FDM	2019	Acrylonitrile butadiene styrene	Carbon fiber	Improved mechanical properties of composites, enhanced printability, and reduction in defects and porosity	[267]
FDM	2018	Polycarbonate (PC), polyamide	Graphene nanoplatelets (GNPs)	Enhanced mechanical and thermal properties of composites, improved printability and surface quality, and cost-effectiveness	[268]
FDM	2017	Nylon, polycarbonate, and ABS	Carbon, glass, and aramid fibers	Enhanced mechanical and thermal properties of composites, improved printability and surface quality, and cost-effectiveness	[269]
FDM	2021	ABS, nylon, and polycarbonate	Carbon, glass, and aramid fibers	Enhanced mechanical and thermal properties of composites, improved printability and surface quality, and cost-effectiveness	[270]
FDM	2023	Polylactic acid (PLA), ABS	Nanoclays and carbon nanotubes	Improved mechanical, thermal, and electrical properties of nanocomposites, enhanced printability, and reduction in defects and porosity	[271]
Inkjet printing	2017	Hydrogels		Customizable shapes, high biocompatibility and cell viability, and the ability to print living tissues and organs	[272]
SLS	2010	Polycaprolactone (PCL)	Hydroxyapatite (HA)	Improved biocompatibility and mechanical properties for tissue engineering applications	[273]
SLA	2022	Polyethylene glycol diacrylate (PEGDA)	Copper nanoparticles	Enhanced antibacterial properties for biomedical applications	[274]
SLA	2022	GelMA/PCL-MA hybrid resins		Enhanced wound healing and antibacterial properties for tissue engineering applications	[275]
SLS	2020	Polycaprolactone (PCL)		Improved biocompatibility and mechanical properties for tissue engineering applications	[276]
SLA	2020	PMMA	TiO_2_ nanoparticles	Enhanced antibacterial properties for biomedical applications	[277]
DIW	2022	Gelatin methacryloyl (GelMA)	Silver nanoparticles	Musculoskeletal tissue regeneration	[278]
DIW	2021	GelMA	CeO_2_/N-halamine hybrid nanoparticles (NPs)	Enhanced wound healing and antibacterial properties	[279]
3D bioprinter	2023	Hydrogel	Sodium alginate (SA)	Antibacterial activity and biocompatibility	[280]
SLS	2022	Polyether ether ketone (PEEK)		Biocompatible, high temperature resistance, and excellent mechanical properties	[281]
SLS	2015	Polycaprolactone (PCL)		Enhanced mechanical and thermal properties of composites, improved printability and surface quality, and cost-effectiveness	[282]
FDM	2018	Poly lactic acid (PLA)		Biodegradable, low-cost, and ease of processing	[283]
FDM	2014	Acrylonitrile butadiene styrene (ABS)		High strength, durability, and ease of processing	[284]

**Table 2:** Summary of the benefits and drawbacks of numerous printing techniques.

Technique	Materials	Application	Benefits	Drawbacks
Fused deposition modeling (FDM)	Poly(lactic acid) (PLA), Polyethylene glycol (PEG), Polyethylene oxide (PEO), etc.	Customized implants, surgical guides, prosthetics, etc.	Low-cost, versatile, and easy to use	Limited strength and stiffness, poor resolution, and surface finish
Stereolithography (SLA)	Photopolymerizable resins, such as acrylates, epoxies, and polyurethanes	Dental models, prosthetics, surgical guides, etc.	High resolution, smooth surface finish, and accuracy	Expensive, limited material selection, and potentially toxic photoinitiators
Selective laser sintering (SLS)	Polyamide (PA), polycarbonate (PC), polyetherimide (PEI), etc.	Customized implants, surgical tools, prosthetics, etc.	High strength, durability, and complex geometries	Expensive, limited resolution, and surface finish
Inkjet printing (IJP)	Hydrogels, synthetic polymers, bioinks, etc.	Tissue engineering, drug delivery, and regenerative medicine	High flexibility, scalability, and control over composition	Limited mechanical properties, resolution, and stability
Electrospinning (ESP)	Polycaprolactone (PCL), polyvinyl alcohol (PVA), collagen, etc.	Tissue engineering, wound healing, and drug delivery	High porosity, biocompatibility, and fiber diameter control	Limited mechanical strength and complex 3D structures
Digital light processing (DLP)	Poly(ethylene glycol) diacrylate (PEGDA), methacrylated gelatin (GelMA), polyurethane (PU), etc.	Tissue engineering, drug delivery, and surgical planning	High resolution, accuracy, and speed	Limited material selection, biocompatibility concerns, and light scattering in thick structures

## Data Availability

The data presented in this study are available on request from the corresponding author.

## Abbreviations

Additive manufacturing	AM
3D printing	3DP
Rapid prototyping	RP
Tissue engineering	TE
Extracellular matrix	ECM
Poly-caprolactone	PCL
Poly-glycolic acid	PGA
Poly(hydroxy butyrate)	PHB
Acrylonitrile butadiene styrene	ABS
Tricalcium phosphate	TCP
Hydroxyapatite	HAP
Computer tomography	CT
Polyethylene glycol dimethacrylate	PEGDMA
Polyetherimide	PEI
Polyether ether ketone	PEEK
Fused filament fabrication	FFF
Acrylonitrile butadiene styrene	ABS
Selective laser sintering	SLS
Biodegradable stents	BRS
Poly-l-lactic acid	PLLA
Methacrylic	MAA
Poly(lactic-co-glycolic acid)	PLGA
Polycaprolactone	PCL
Smooth muscle cells	SMCs
Endothelial cell layer	ECs
Poly-3,4-ethylenedioxythiophene	PEDOT
Polystyrene sulfonate	PSS
Critical micelle concentration	CMC
Polyethene oxide	PEO
Polyurethane	PU
Bovine aortic endothelial cells	BAECs
Tissue engineering	TE
Two-photon polymerization	2PP
